# Boundary Lubrication Performance of Polyelectrolyte–Surfactant Complexes on Biomimetic Surfaces

**DOI:** 10.1021/acs.langmuir.3c03737

**Published:** 2024-04-04

**Authors:** Erik Weiand, Peter H. Koenig, Francisco Rodriguez-Ropero, Yuri Roiter, Stefano Angioletti-Uberti, Daniele Dini, James P. Ewen

**Affiliations:** † Department of Mechanical Engineering, Imperial College London, South Kensington Campus, London SW7 2AZ, U.K.; ‡ Institute of Molecular Science and Engineering, Imperial College London, South Kensington Campus, London SW7 2AZ, U.K.; § Thomas Young Centre for the Theory and Simulation of Materials, Imperial College London, South Kensington Campus, London SW7 2AZ, U.K.; ∥ Corporate Functions Analytical and Data & Modeling Sciences, Mason Business Center, The Procter and Gamble Company, Mason, Ohio 45040, United States; ⊥ Department of Materials, Imperial College London, South Kensington Campus, London SW7 2AZ, U.K.

## Abstract

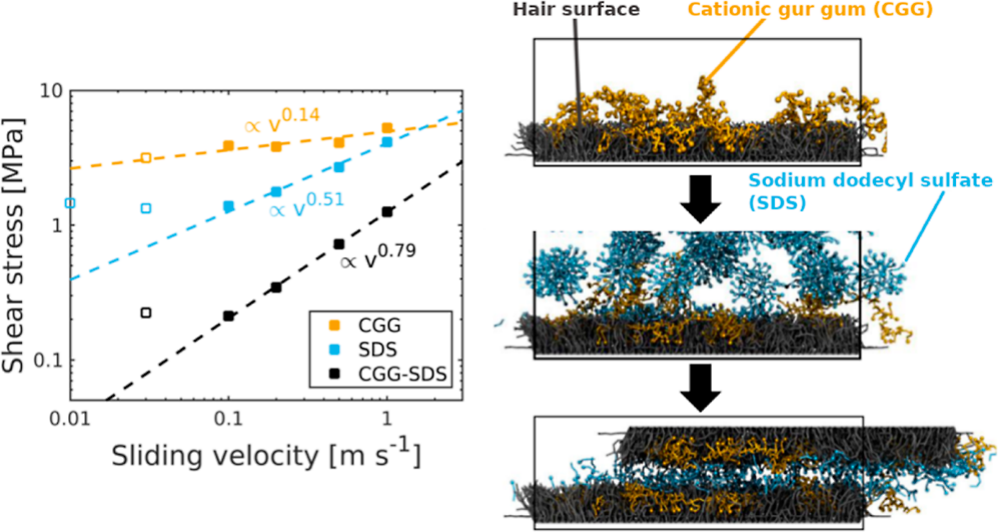

Aqueous mixtures of oppositely charged polyelectrolytes and surfactants are useful in many industrial applications, such as shampoos and hair conditioners. In this work, we investigate the friction between biomimetic hair surfaces in the presence of adsorbed complexes formed from cationic polyelectrolytes and anionic surfactants in an aqueous solution. We apply nonequilibrium molecular dynamics (NEMD) simulations using the coarse-grained MARTINI model. We first developed new MARTINI parameters for cationic guar gum (CGG), a functionalized, plant-derived polysaccharide. The complexation of CGG and the anionic surfactant sodium dodecyl sulfate (SDS) on virgin and chemically damaged biomimetic hair surfaces was studied using a sequential adsorption approach. We then carried out squeeze-out and sliding NEMD simulations to assess the boundary lubrication performance of the CGG–SDS complex compressed between two hair surfaces. At low pressure, we observe a synergistic friction behavior for the CGG–SDS complex, which gives lower shear stress than either pure CGG or SDS. Here, friction is dominated by viscous dissipation in an interfacial layer comprising SDS and water. At higher pressures, which are probably beyond those usually experienced during hair manipulation, SDS and water are squeezed out, and friction increases due to interdigitation. The outcomes of this work are expected to be beneficial to fine-tune and screen sustainable hair care formulations to provide low friction and therefore a smooth feel and reduced entanglement.

## Introduction

Shampoos and conditioners contain a wide range of surfactants and polymers to facilitate the cleansing and conditioning of hair.^1,2^ The outer surface of virgin hair is covered by a fatty acid monolayer, mostly made up of 18-methyleicosanoic acid (18-MEA), which makes it hydrophobic.^3^ When hair is chemically damaged, such as through bleaching, the fatty acid molecules are removed and the underlying cysteine protein layer is oxidized to yield anionic cysteic acid groups, which makes them hydrophilic.^3^ The exposure of anionic groups also leads to higher friction of bleached hair compared to virgin hair.^4^ Therefore, a key function of shampoos and particularly conditioners is to temporarily repair this damage.^2,5^ This is usually achieved using cationic surfactants or polyelectrolytes, which strongly adsorb onto the damaged regions on the hair surface.^6,7^ Indeed, the adsorption of cationic surfactants and polyelectrolytes on bleached hair has been shown to restore both the low friction^8−11^ and the hydrophobic^11−16^ properties of virgin hair. On the other hand, the primary role of anionic surfactants in hair care formulations is to remove dirt particles and sebum from the hair surface.^17^ They do this by forming micelles at relatively low concentrations, which can trap oily substances in their core.^17^ Since anionic surfactants adsorb only weakly on the negatively charged hair surface through hydrophobic interactions,^11^ they can readily desorb from the hair surface to remove the trapped substances. Thus, most shampoos now contain both cationic polyelectrolytes for conditioning and anionic surfactants for cleansing.^17^

The addition of ionic surfactants to aqueous solutions of polyelectrolytes with the opposite charge can cause the spontaneous formation of supramolecular complexes.^18^ These polyelectrolyte–surfactant complexes are important to the function of many formulated consumer products such as cosmetics, pharmaceuticals, and foods.^19^ Depending on the surfactant concentration, the complexes can either remain soluble in water or undergo liquid–liquid (coacervation) or liquid–solid (precipitation) phase separation, which can be utilized to deposit complexes onto solid surfaces by dilution.^19^ In hair care products, this behavior is used to promote the deposition of insoluble cationic polymers onto the hair surface during rinsing.^20^ This process can also be used to aid the deposition of other beneficiary agents onto hair, most commonly silicones, which are used for their conditioning properties.^21^ The formation of polyelectrolyte–surfactant complexes can be beneficial to the bulk properties of the hair care product such as enhancements of viscosity, foaming or gelling performance.^22^ Once deposited on the hair surface, polyelectrolyte–surfactant complexes can significantly reduce friction and stick–slip between hairs.^23−26^ Several factors, such as the surfactant and salt concentration, as well as the polyelectrolyte charge density and molecular weight, can affect the amount of polyelectrolyte deposited and its conformation on the hair surfaces.^27−29^ This has direct consequences for the lubrication performance for polyelectrolyte–surfactant complexes.^30^ Despite its importance to a wide range of products, the boundary lubrication mechanism of polyelectrolyte–surfactant complexes remains mostly unknown.^31^

More environmentally friendly alternatives to petroleum-based feedstocks are urgently being sought by hair care formulators. These alternatives can be derived from natural sources, but should provide an equal or superior performance.^32^ One promising class of sustainable polymers are polysaccharides,^1^ such as guar gum, which can be extracted from the seeds of the guar plant.^33^ Guar gum is used extensively in food products as a texture and rheology modifier and its derivatives, such as cationic guar gum (CGG), have become increasingly important in several industries, such as oil and gas extraction as well as textile and paper processing.^34^ CGG is known to be effective in reducing the friction between hairs and the addition of anionic surfactants, such as sodium dodecyl sulfonate (SDS), can further improve its lubrication performance.^23^

Theory and molecular modeling have provided a valuable complement to experiments to resolve the structure and friction of adsorbed polyelectrolyte–surfactant complexes. The self-assembly and aggregation of oppositely charged polyelectrolyte–surfactant complexes in water has been investigated using Monte Carlo,^35^ dissipative particle dynamics (DPD),^36^ and molecular dynamics (MD) simulations.^37,38^ Self-consistent field theory (SCFT) has also been used to study the self-assembly of polyelectrolyte–surfactant complexes and their adsorption to biomimetic surfaces.^39−41^ Recently, nonequilibrium molecular dynamics (NEMD) simulations have been used to investigate the structure and friction of oppositely charged polyelectrolyte–surfactant complexes inside hair contacts.^42^ These methods provide the opportunity to virtually screen polyelectrolytes and surfactants for optimal friction performance on biomimetic hair surfaces.^1^

In this study, we use NEMD simulations to investigate the friction of biomimetic hair surfaces lubricated by complexes formed from CGG and SDS. As with our previous MD and NEMD simulation studies of hair wettability and friction,^4,11,43^ we employ the coarse-grained MARTINI 2 force field.^44,45^ We first develop new parameters for CGG using a bottom-up approach from atomistic MD simulations. We then perform sequential adsorption simulations of CGG and then SDS on biomimetic surfaces representative of virgin and chemically damaged hair. Squeeze-out simulations are used to determine the composition within the contact at physiologically relevant pressures. Finally, NEMD simulations are performed to probe the friction between two hairs with adsorbed polyelectrolyte–surfactant complexes at various physiologically relevant sliding speeds and pressures. The framework presented in this study could be applied to virtually screen the lubrication performance of a wide range of hair care formulations on biomimetic surfaces. By changing the surface model, the methodology can be readily extended to investigate other formulated products that contain polymer–surfactant complexes, such as in fabric softeners^46^ or for drug delivery.^47^

## Materials and Methods

### Polyelectrolyte Model

**Figure 1:**
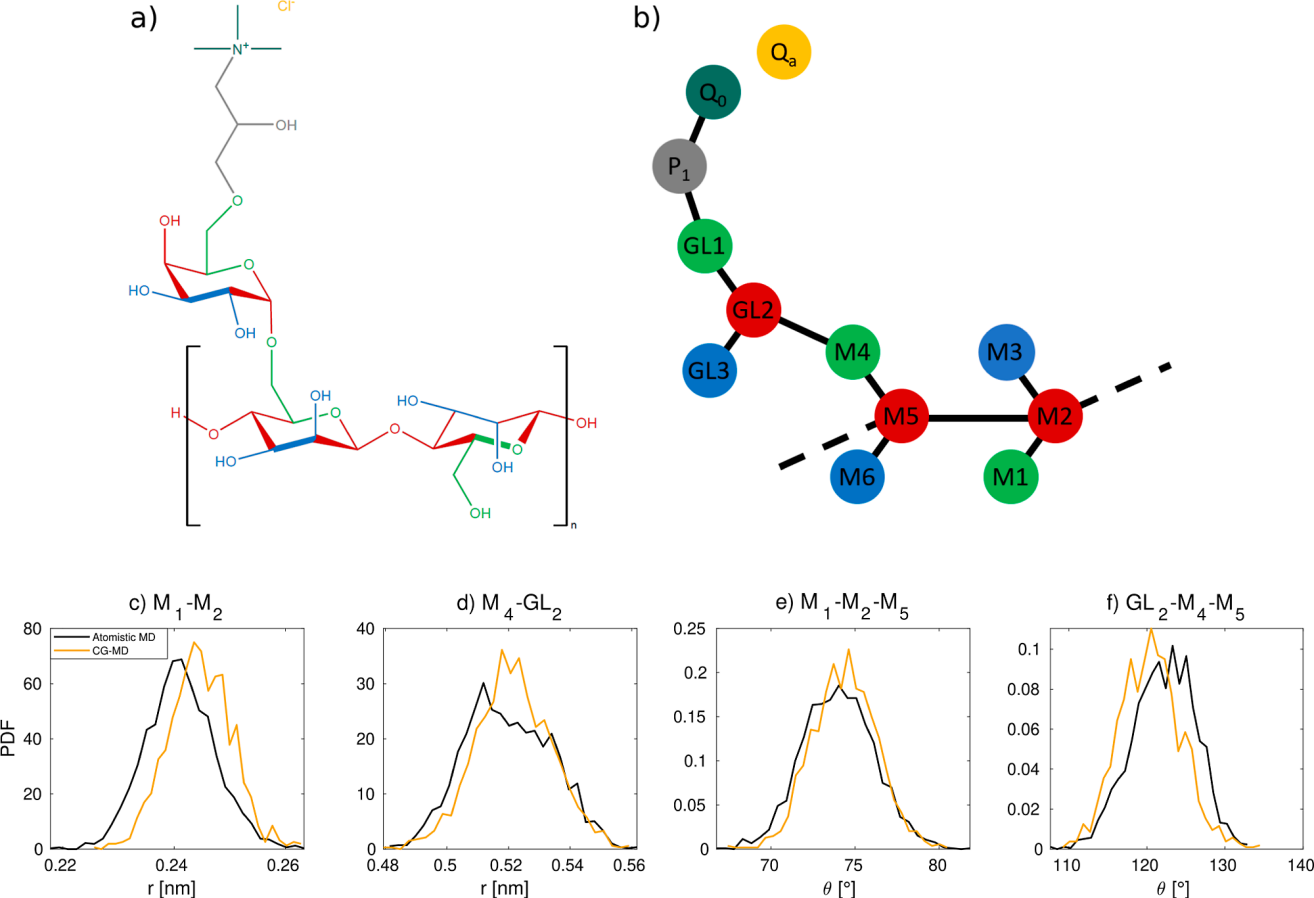
(a) chemical structure of guar hydroxypropyltrimonium chloride and (b) the corresponding coarse-grained architecture. The coarse-grained color coding for carbohydrate groups is in accordance with ref (48). Examples of the mapping of bond and angle potential between atomistic and coarse-grained simulations for guar gum are shown in (c–f).

For the polyelectrolyte, we selected CGG, or more specifically guar hydroxypropyltrimonium chloride, which is a quaternary ammonium-functionalized polysaccharide with high-molecular weight (*M*_w_ = 1,000,000–3,000,000 g mol^–1^)^33,49,50^ and promising friction properties on hair.^23^Figure 1a shows the structural formula of the guar hydroxypropyltrimonium chloride monomer. The backbone consists of two β-d-mannopyranose units with an α-d-galactose branch functionalized with a quaternary ammonium group.^33^

The MARTINI force field^44,45^ has been used to study a wide range of oppositely charged polyelectrolytes and surfactants in bulk^51−54^ and in the presence of surfaces.^42^ MARTINI typically uses a 4:1 mapping of heavy atoms to coarse-grained beads.^44,45^ The first MARTINI 2 parametrization for saccharides was developed by López et al.,^55^ who established a set of building blocks required for parametrizing a wide range of polysaccharides. Shivgan et al.^48^ proposed a significantly different mapping to that proposed by López et al.,^55^ which overcame the overestimated aggregation propensity of polysaccharides using this model.^56^ Xu and Matysiak^51^ presented a coarse-grained model of the polysaccharide chitosan using the polarizable MARTINI water model due to Yesylevskyy et al.,^57^ which was recently used by Gotla et al.^52^ to study chitosan interactions with SDS. Tsanai et al.^53^ and Liu et al.^54^ used MARTINI 3^58^ to investigate the salt-dependent coacervation of oppositely charged polyeletrolyes. MARTINI 2 with the polarizable MARTINI water model has also been used to study polelectrolyte complexes, such as those formed between poly(styrenesulfonate) and poly(diallyldimethylammonium).^59^ A more heavily coarse-grained force field for guar was proposed by Liang et al.^60^ in which one bead was used for each monosaccharide (∼10 heavy atoms), resulting in 3 beads per repeat unit, as opposed to the 9 used in MARTINI. However, to our knowledge, no parametrization for guar or its cationic derivatives is currently available in the MARTINI framework. Therefore, we first generate new MARTINI 2^44,45^ parameters for guar and guar hydroxypropyltrimonium chloride. We chose MARTINI 2^44^ over MARTINI 3^58^ due to the significant amount of previous validation work we have performed for hair surfaces using this model^4,11,43^ and the availability of a polarizable water model.^57^ The parameters are determined using a bottom-up approach from atomistic MD simulations.

### Atomistic MD Simulations

All of the atomistic and coarse-grained MD simulations in this work were conducted using the open-source large-scale atomic/molecular massively parallel simulator (LAMMPS) software.^61^ Atomistic MD simulations of solvated guar and cationic-modified guar monomers and short oligomers were conducted using the OPLA-AA force field,^62^ which has been extensively validated for polysaccharides.^63,64^ Initial atomistic topologies for nonionic and cationic guar were obtained from LigParGen.^65−67^ The monomers and short oligomers were solvated in 4180 SPC/E water molecules^68^ in a fully periodic simulation box. The Lennard-Jones (LJ) interactions were cut off at a distance of 1.2 nm. Long-range Coulombic interactions are calculated using the particle–particle, particle-mesh (PPPM) method^69^ with a relative energy tolerance of 10^–5^. The preparation of bulk systems was established with the open-source codes Packmol^70^ and Moltemplate.^71^ The system was energy minimized and subsequently thermostated at a temperature of 300 K and barostatted at a pressure of 1 atm in the isothermal–isobaric (*NPT*) ensemble using a Nosé–Hoover thermostat^72,73^ and barostat^74^ with damping constants of 0.1 ps for temperature and 1 ps for pressure. An integration time step of 2 fs was applied while constraining all hydrogen bonds using the SHAKE algorithm.^75^ The equations of motion were integrated using the velocity-Verlet algorithm.^76^ After energy minimization and an equilibration run of 1 ns, monomer trajectories were recorded every 2 ps for a duration of 4 ns.

### Coarse-Grained MD Simulations

#### Guar and CGG MARTINI Parametrization

The coarse-grained mapping of guar hydroxypropyltrimonium monomers is shown in Figure 1a,b. A modified Boltzmann inversion technique is used via the open-source PyCGTOOL software^77^ to obtain bonded interaction parameters for the coarse-grained molecule based on the trajectories from the atomistic MD simulations. The MARTINI parameters are generated from atomistic simulation trajectories, rather than fitting to a single snapshot and therefore provide a more accurate representation of the distribution of bond lengths and angles from the atomistic reference models.^77^ The GROMACS files are then converted to LAMMPS files using GRO2LAM.^78^ We developed parameters for both nonfunctionalized guar monomers and cationic-functionalized guar monomers to enable a varying degree of substitution (DS) and thus polyelectrolyte charge density. The guar mapping is identical to that in Figure 1b, but without the hydroxypropyltrimonium group. The nonbonded LJ interactions for the mannose and galactose building blocks in guar are obtained from previous MARTINI 2 parametrizations of polysaccharides by Shivgan et al.^48^ Unlike Shivgan et al.,^48^ we do not downscale the LJ interactions to maintain consistency with our validated unscaled MARTINI LJ parameters for hair surfaces.^4,11,43^ The nonbonded LJ interactions for the trimethylammonium ion (Q_0_ bead, which carries a permanent charge of +1*e*) and chloride counterion (Q_a_ bead, which carries a permanent charge of −1*e*), as commonly employed for cationic surfactants.^79^ For the hydroxypropyl group, we use a P_1_ bead, which is the standard alcohol group in MARTINI 2.^44^ The nonbonded LJ interactions from the polarizable water model due to Yesylevskyy et al.^57^ were used without modification. Therefore, we developed new parameters for the bonds and angles between the beads. Dihedral interactions were omitted after initial tests showed reduced numerical stability during the MD simulations, as is commonly observed when using the MARTINI model^45^ and other coarse-grained force fields.^60^ The full list of MARTINI 2 parameters for both guar and guar hydroxypropyltrimonium chloride is provided in the Supporting Information (Table S1). Figure 1c–f shows four examples of the mapped harmonic bond and angle potentials from atomistic and coarse-grained bulk simulations of cationic guar in an aqueous environment. Comparisons of the other bonds and angles parametrized here are shown in the Supporting Information (Figures S2 and S3).

A comparison of the increase in the radius of gyration, *R*_g_, of neutral guar with molecular weight, *M*_w_, between MARTINI and experiments is shown in the Supporting Information (Figure S4). At a given *M*_w_, our MARTINI parameters consistently underestimate the absolute values of *R*_g_ compared to previous scattering experiments.^80^ This discrepancy could be attributed to the high polydisperisty of the experimental samples, since higher *M*_w_ species are emphasized in scattering experiments.^60^ The *R*_g_ follows the power-law scaling law ∝*M*_w_^0.62^, which is in good agreement with previous experiments (∝*M*_w_^0.62^)^80^ and coarse-grained MD simulations (∝*M*_w_^0.60^).^60^

The linear CGG polymer chains were constructed according to a self-avoiding random walk using the genpoly extension available in Moltemplate.^71^ The degree of substitution, DS, which represents the fraction of monomers functionalized with a cationic group, can vary between around 4–30% for guar hydroxypropyltrimonium chloride.^49,81^ In this work, we consider cationic guar gum at a maximum charge density (DS = 100%). Higher charge densities are potentially interesting for industrial formulations due to their stronger affinity toward negatively charged surfactant molecules and hair substrates.^24^ A molecular weight of *M*_w_ = 36,900 g mol^–1^ was chosen for our monodisperse CGG, which corresponds to 50 repeat units. This is much shorter (1–4%) than the reported values for industrial cationic guar derivatives (*M*_w_ = 1,000,000–3,000,000 g mol^–1^),^49^ but allows for system dimensions which are computationally accessible.^42^ The application of shear is expected to lead to stretching and preferential orientation of the polymers in the direction of sliding, as shown in previous coarse-grained NEMD simulations of polymers on biomimetic hair surfaces.^82^ Such extended polymers with an end-to-end distance larger than the dimensions of the simulation box could cause unphysical self-interactions across periodic images during sliding. Thus, a relatively short polymer size is necessary to facilitate a computationally tractable system size. The systems used for the NEMD simulations in this work contain approximately 75,000 coarse-grained beads, while some squeeze-out simulations contain more than 500,000 beads.

#### Adsorption of CGG & SDS

**Figure 2:**
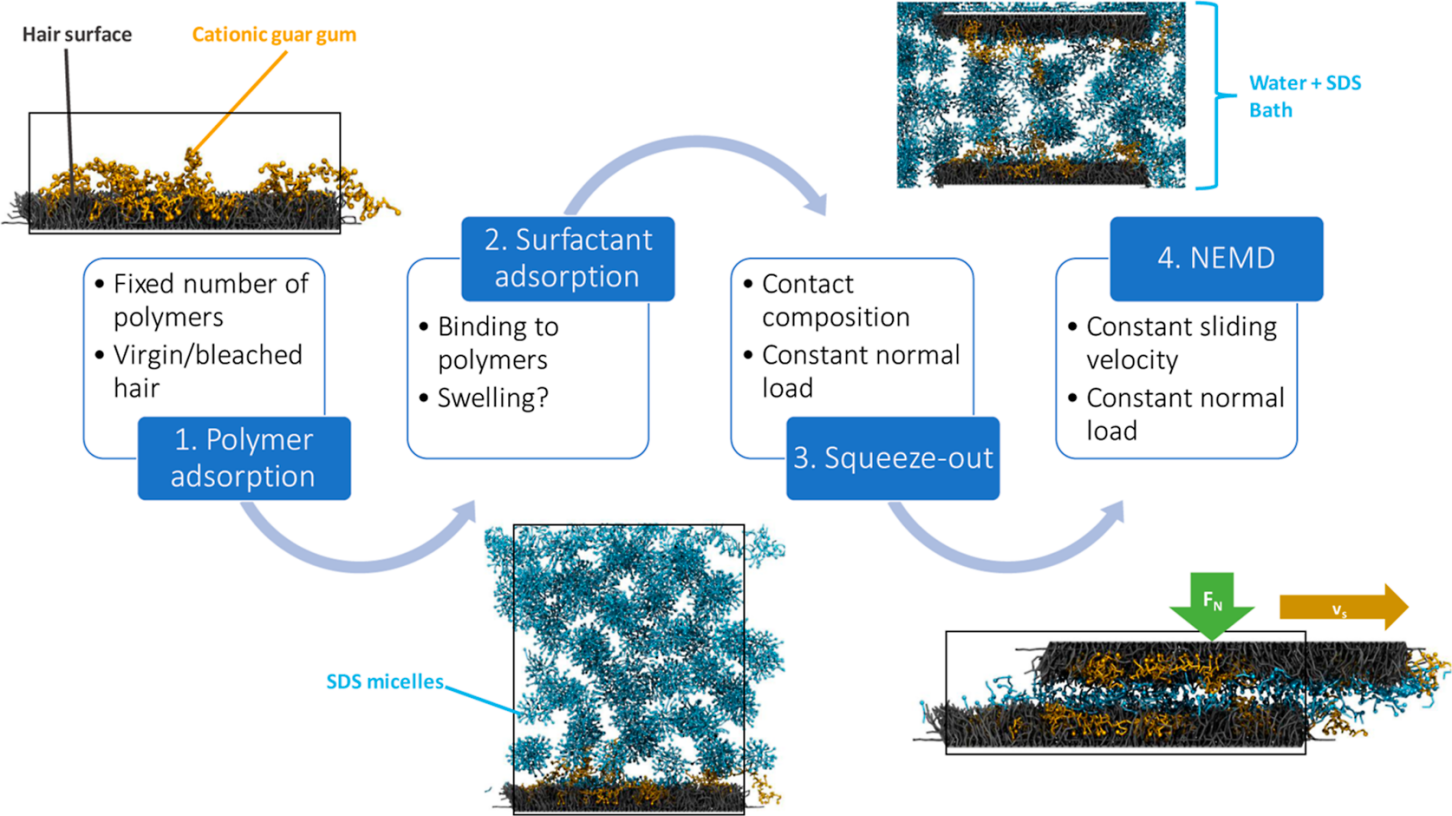
Polyelectrolyte–surfactant lubrication workflow consisting of sequential adsorption of CGG and then SDS, followed by squeeze-out simulations to determine the contact composition and finally NEMD simulations.

To study friction of polymers and surfactants at hair surfaces, we apply a workflow consisting of sequential adsorption, squeeze-out, and NEMD simulations, as summarized in Figure 2. Adsorption of cationic guar gum and anionic surfactants to biomimetic hair surfaces is studied using an existing model of the outer layer of virgin and medium bleached hair.^43^ These biomimetic surface models have been extensively validated against experiments for various properties such as contact angle, roughness, surface energy^11,43^ and friction.^4^ In this study, we establish the polymers and surfactants on the surface by means of sequential adsorption, where cationic polyelectrolytes are adsorbed first, followed by adsorption simulations of anionic surfactants from a micellar bulk. The adsorption of self-assembled polymer–surfactant complexes, as recently studied by Coscia et al.^42^ is also of interest but requires fundamental changes of our proposed routine to establish the amount of water, salt and anionic surfactants at the interface (squeeze-out simulations). We justify the sequential adsorption procedure pursued in this work by three possible scenarios on real hair surfaces:

(1) Fast adsorption of cationic polymers from the bulk compared to surfactants due to the long-range electrostatic interactions (∝*r*^–2^) with the oppositely charged surface charges.^83^ Previous SCFT calculations^39,40^ reported a similar layered adsorption structure for branched cationic polyelectrolytes with anionic surfactants on biomimetic hair surfaces.
(2) In some situations, cationic polymers are already present on the hair surface before treatment with other products containing anionic surfactants, for example conditioned hair that is washed with a shampoo.^84^
(3) Even in experiments, adsorbed polyelectrolyte–surfactant complexes can be trapped in quasi-equilibrium states^85^ and the true equilibrium may only be reached after time scales inaccessible to our simulations (days).^86^

The sequential adsorption simulation protocol proposed here also allows for a direct observation of potential SDS-induced swelling effects of the cationic polymer on the surface, as observed in previous ellipsometry measurements on hydrophilic silica.^87^ The approach was also previously used in adsorption studies of cationic polysaccharides on silica which where then gradually exposed to increasing SDS concentrations.^88,89^ It is worth noting that the polyelectrolyte–surfactant structures obtained through sequential adsorption might be fundamentally different to those obtained from adsorption of preformed complexes due to kinetic entrapment.^27^

An integration time step of 5 fs was used for all the coarse-grained MD simulations. We use the polarizable MARTINI water model,^57^ which consists of a central LJ bead and two oppositely charged satellite beads. Compared to the nonpolarizable MARTINI water model,^44^ the polarizable model is less prone to freezing under confinement. The bonds in the water model were constrained at an equilibrium bond length of *r* = 0.14 nm using the SHAKE algorithm.^75^ A harmonic angle potential with equilibrium angle, Θ = 0°, and force constant, *K*_Θ_ = 2.1 kJ mol^–1^ rad^–2^ (in LAMMPS, already including a factor of 1/2), is added to control the rotation of the satellite beads.^57^ Nonbonded interactions between coarse-grained beads are considered using shifted LJ potentials.^44^ Smooth shifting of LJ contributions to zero is performed between from *r*_LJ,c_ = 0.9 to *r*_LJ,s_ = 1.2 nm. Long-range Coulombic interactions are calculated using the PPPM method^69^ with a relative energy tolerance of 10^–5^. For the confined systems used in adsorption and sliding simulations, the slab implementation of the PPPM algorithm^90^ was used. Harmonic potentials are applied for the bonds and angles;^44^ the parameters are given in the Supporting Information (Table S1).

Using Moltemplate,^71^ we first create bulk systems, containing 4 or 12 CGG polymers, 200 or 600 hydrated chloride counterions, and 34,299 or 64,310 water beads for low and high guar concentrations, respectively. The total number of coarse-grained beads in the bulk systems is 105,297 for the low CGG concentrations and 200,130 for the high concentration. The guar bulk system is energy minimized by using the conjugate gradient method and subsequently undergoes thermal relaxation at *T* = 600 K and *p* = 1 atm in the *NPT* ensemble. We use the Nosé–Hoover thermostat^72,73^ and barostat^74^ with damping constants of 1 ps for temperature and 3 ps for pressure. Then, the entire bulk system containing the final number of polymers is further thermally relaxed in aqueous solution at *T* = 600 K for 30 ns, before equilibrating for another 5 ns at a target temperature of *T* = 300 K. This equilibrated bulk system is then transferred to above the hair surface for subsequent adsorption simulations. We selected an initial number of CGG polymers on each of the surfaces, *N*_CGG_ = 4, which assuming full adsorption, corresponds to an equilibrium adsorption density of Γ = 0.04 μg cm^–2^. The chosen adsorption density, Γ, is motivated by ellipsometry measurements of the adsorption density of CGG on hydrophobic (virgin hair model), where (Γ = 0.04–0.05 μg cm^–2^) and hydrophilic silica (bleached hair model), where Γ = 0.06 μg cm^–2^ after an adsorption time of 5000 s.^50^ Such time scales are beyond the limits of our coarse-grained MD simulations, so polymers are placed close to the surface to ensure full adsorption at a given coverage. This approach was also used in previous atomistic MD simulations of surfactants adsorbed on iron oxide surfaces.^91^ Dissipative quartz crystal microbalance (D-QCM) adsorption measurements of another cationic polysaccharide, JR400, to uncharged thiol surfaces (virgin hair model) conducted by Guzmán et al.^83^ also indicated similar surface coverages (Γ = 0.05 μg cm^–2^). Higher surface coverages of JR400 Γ ≈ 0.12 μg cm^–2^ have been observed by D-QCM measurements of JR400 on negatively charged thiol monolayers (bleached hair model).^83^ The use of biomimetic surfaces, rather than real hair for these experimental measurements, eliminates the need to convert adsorption densities that are typically given as a fraction of the weight of the measured hair sample, thus requiring an accurate measurement of the surface area per unit weight.^92^ The effect of an increasing adsorption density is expected to be particularly relevant for bleached hair, where a comparable increase in the charge density is expected.

An equilibrated system containing anionic surfactants, specifically SDS molecules *N*_SDS_ = 4, 328, are then introduced on top of the existing surfaces with adsorbed CGG at an initial concentration of *c*_SDS_ = 664 mM and a finite system height of Δ*z* = 21 nm. Standard MARTINI 2 parameters for SDS are employed, with three apolar C_1_ beads attached to one Q_a_ (−1*e*) bead, with a Q_d_ (+1*e*) bead for the hydrated sodium counterion.^93^ In commercial shampoos, the anionic surfactant SLES is generally present at concentrations of between 50 and 150 mM.^17^ Similar SDS and SLES concentrations were also used in previous friction experiments of complexes formed with cationic guar.^23,84^ The selected concentration is well above the critical micelle concentration (CMC) of SDS *c*_cmc_ = 8.2 mM,^94^ so the molecules are predominantly present as cylindrical micelles.^93^ The high SDS concentration was chosen to obtain a strong local excess of anionic sites (22:1), which was required to obtain binding of micellar SDS to guar within reasonable computational times.^93^ Either coarse-grained sodium (Na^+^) or chloride (Cl^–^) counterions with their first hydration shell^45^ are added to the CGG and SDS bulk systems to ensure charge neutrality during adsorption. No excess salt was considered in these simulations. Our previous study showed that excess salt reduced the propensity for adsorption of cationic surfactants on models for virgin and bleached hair.^11^

The *x* × *y* dimensions of the virgin and bleached hair surfaces are 24 × 21 nm for the adsorption simulations. The adsorption simulations are conducted with periodic boundary conditions applied in the *x* and *y* directions. These systems are finite in the *z* direction and a repulsive boundary is added at the top of the simulation cell.^11,95^ Simulations are run at a temperature of *T* = 300 K for a maximum duration of 50 ns. The temperature is controlled with a Nosé–Hoover thermostat^72,73^ with a damping constant of 1 ps.

#### Squeeze-Out and NEMD

Squeeze-out MD simulations are conducted between two hair surfaces following the protocols we previously developed for water between biomimetic hair^4,11^ and *n*-hexadecane between iron oxide substrates.^91^ Contacts are created by replicating and reflecting the surfaces containing CGG and SDS from the adsorption simulations, with an initial separation distance of 24 nm. The contact is immersed in an equilibrated micellar SDS solution (*c*_SDS_ = 664 mM), as shown in Figure 2. The *x* × *y* × *z* dimensions of the systems are 40 × 21 × 32 nm for the squeeze-out simulations, with periodic boundary conditions applied in all directions. The contact length in the *y* direction is equal to the box length in *y* and is, therefore, periodic in this direction. During compression, water and SDS molecules are squeezed out into the reservoir space. Compression between the two hair surfaces was achieved by applying a constant normal load (σ = 5–50 MPa) between the graphene sheets of the opposing hair surfaces. The range of contact pressures σ was established in previous contact mechanics estimates for hair–hair contacts.^4^ The contact thickness was recorded as a function of time and simulations were run until equilibrium contact conditions were established, which was typically within 50–100 ns, as shown in the Supporting Information (Figure S5). At σ = 10 MPa, the equilibrium contact thickness was approximately 6 nm for both virgin and medium bleached hair, which is somewhat thicker than for pure water (4–5 nm).^4^ The CGG polymers remain adsorbed and are not squeezed out due to the multiple cationic groups that form strong ionic bonds with the sulfonate groups on the hair surfaces. Previous experiments have shown that cationic polymers are resistant to squeeze out up to high loads on anionic-modified surfaces.^96^ Similar observations were made previously for cationic surfactants, which were also resistant to squeeze out.^11^

Constant shear-rate NEMD simulations^97^ are then conducted between the two compressed hair surfaces with a contact composition established by the squeeze-out simulations. For the sliding simulations, the external reservoir is removed and the *x* × *y* dimensions of the system are reduced such that they are the same as the hair surfaces (24 × 21 nm). Physiologically relevant constant sliding velocities of *v*_s_ = 0.03–1 m s^–1^ are applied to the anchoring substrate of the upper surface while maintaining the constant normal load from the squeeze-out simulations. This range of velocities is relevant to hair manipulations such as touching, brushing, and combing.^4^ The system temperature is controlled using a Langevin thermostat^98^ applied only in the *y* direction and to the base bead of the surface-grafted molecules (18-MEA and cysteic acid). Further information on the NEMD protocol applied can be found in our previous studies.^4,11^

## Results and Discussion

### CGG–SDS Adsorption

#### Sequential Adsorption

We first perform adsorption simulations of CGG at a degree of substitution, DS = 100%, onto biomimetic virgin and medium bleached hair surfaces. There is a strong electrostatic affinity between the cationic groups on CGG and the negatively charged sites on the hair surfaces. This means that the CGG polymers lie mostly flat on the surface, which is consistent with previous experiments for high-charge density polyelectrolytes.^99^ We define the thickness of the CGG film as the distance in the surface-normal direction containing 98% of the CGG mass (between 1 and 99% of the time-averaged cumulative mass density distribution). For the pure CGG systems (Figure S6), we measure film thicknesses of 4.4 nm for virgin and 2.9 nm for bleached hair surfaces. In previous experiments, the thickness of CGG layers on hydrophilic silica was reported as around 19 nm.^50^ This discrepancy is probably due to (i) the much larger CGG *M*_w_ in the experiments (around 2,000,000 g mol^–1^) than the simulations (36,900 g mol^–1^) and (ii) the high charge density used in the simulations. Previous studies have shown that increasing the charge density of CGG resulted in a more compact layer.^81^ The mass fraction of water in the CGG film is 65% for virgin hair and 72% for bleached hair. The observed hydration levels are slightly lower than that observed in combined D-QCM and ellipsometry measurements of another cationic polysaccharide (JR400) adsorbed onto surfaces functionalized with anionic groups (75%).^83^ It should be noted that JR400 has a different polysaccharide backbone and lower charge density (DS = 27%) compared with the CGG simulated here (DS = 100%). Previous experimental studies have suggested that more highly charged polyelectrolytes result in less hydrated layers than those obtained from low charged polyelectrolytes.^100^ Thus, the small deviation in polyelectrolyte hydration between the experiments^83^ and current simulations can be attributed to the differences in charge density.

**Figure 3:**
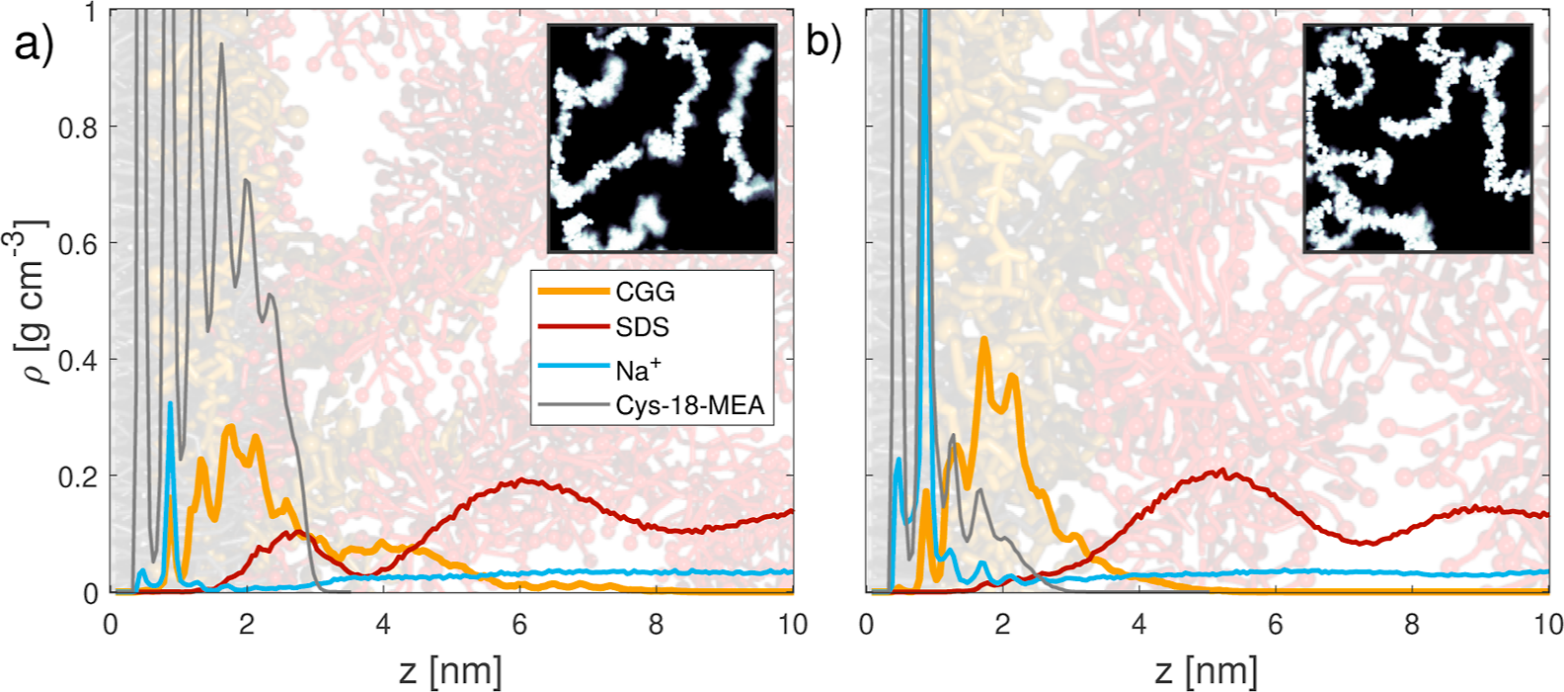
Mass density profiles for (a) virgin and (b) medium bleached hair at the end of the SDS adsorption stage for a given guar adsorption density of Γ = 0.04 μg cm^–2^. Water densities are not shown for clarity. Snapshots show the corresponding systems with CGG beads shown in orange, SDS in red, Na^+^ in blue, and 18-MEA in gray. Insets show the top-view distribution of cationic guar on the surfaces.

Adsorption of micellar SDS is then studied in a second step. Figure 3 shows two simulation snapshots overlaid with equilibrium mass density profiles of the CGG and SDS adsorption structures on the virgin and medium bleached hair surfaces. SDS predominantly adsorbs as intact micelles that bind to the cationic sites on the CGG chains, which are not adsorbed on the surface. On the virgin hair surface, some SDS molecules also bind through hydrophobic adsorption to the tail beads in the 18-MEA layer on the surface. Hydrophobic adsorption was also observed previously in coarse-grained MD adsorption simulations of SDS on untreated virgin hair surfaces.^11^

When SDS is added, the CGG film thickness on virgin hair increases to *d* = 6.3 nm, which is due to swelling. Compared to the pure CGG systems (Figure S6), more of the polymers are in loop or tail conformations, but the majority remain in flat train conformations.^82^ Tails and loops have previously been postulated to be important for the lubrication performance of CGG.^84^ Previous experiments observed swelling of the CGG layers up to 100 nm at high concentrations on functionalized anionic silica.^50^ The reduced swelling in our simulations can again probably be related to the relatively short polymers. Another factor is that in the experiments, the surface charge is usually overcompensated by the oppositely charged adsorbed polyelectrolyte.^96^ In the systems studied in our simulations, the surface charge is not overcompensated, as shown in Table 1 (surface/CGG charge ratio <1 for overcompensation). The remaining fraction of anionic sites on the surface is locally neutralized by sodium counterions. Therefore, there are fewer free cationic groups available to adsorb the SDS micelles and cause swelling. Higher CGG surface coverages would lead to overcompensation of the surface charge, which may lead to increased swelling and improved lubrication.^84^

**Table 1:** Overview of the Contact Composition Used in the NEMD Simulations on Virgin and Medium Bleached Hair Surfaces at Contact Pressures of σ = 5–50 MPa^a^

Virgin hair	σ =	5 MPa	**10 MPa**	15 MPa	20 MPa	35 MPa	50 MPa
Coarse-grained water	ρ_w_	23.1 nm^–^^2^	**16.9 nm^−2^**	11.1 nm^–^^2^	10.1 nm^–^^2^	8.8 nm^–^^2^	7.7 nm^–^^2^
SDS	ρ_SDS_	1.52 nm^–^^2^	**1.11 nm^−2^**	1.15 nm^–^^2^	0.96 nm^–^^2^	0.73 nm^–^^2^	0.66 nm^–^^2^
SDS/CGG charge ratio	*R*_SDS_	1.88	**1.37**	1.41	1.18	0.90	0.81
Hair/CGG charge ratio	|*R*_surf_|	1.68	**1.68**	1.68	1.68	1.68	1.68

Medium bleached hair	σ =	5 MPa	**10 MPa**	15 MPa	20 MPa	35 MPa	50 MPa
Coarse-grained water	ρ_w_	28.1 nm^–^^2^	**20.9 nm**^**−2**^	18.2 nm^–^^2^	16.8 nm^–^^2^	13.4 nm^–^^2^	12.9 nm^–^^2^
SDS	ρ_SDS_	1.04 nm^–^^2^	**0.87 nm**^**−2**^	0.80 nm^–^^2^	0.69 nm^–^^2^	0.57 nm^–^^2^	0.54 nm^–^^2^
SDS/CGG charge ratio	*R*_SDS_	1.28	**1.07**	0.98	0.85	0.70	0.66
Hair/CGG charge ratio	|*R*_surf_|	5.62	**5.62**	5.62	5.62	5.62	5.62

a The initial surface area density of CGG chains, ρ_CGG_ = 0.016 nm^–2^, remained unchanged during squeeze-out. The baseline case at σ = 10 MPa is shown in bold.

When SDS is added to the system with CGG adsorbed on medium bleached hair, most of the polymers remain in a train conformation on the surface.^82^ There is some swelling upon addition of SDS, with the CGG layer extending to 3.8 nm. Swelling is less pronounced on bleached hair than virgin hair due to the higher negative surface charge density. The mass fraction of water in the CGG films is 64% for virgin hair and 65% for medium bleached hair. Thus, the CGG films remain highly hydrated upon adsorption of SDS, which may be important for effective lubrication.^24^ The similarity in the degree of hydration for the CGG on virgin and medium bleached surfaces, despite the higher CGG film thickness on virgin hair, can be explained by the volume exclusion due to the higher 18-MEA coverage on virgin hair. The insets in Figure 3 show that CGG polymers are distributed heterogeneously on the surfaces in stretched conformations, which are similar to those observed experimentally for polyelectrolytes adsorbed on oppositely charged surfaces.^101^ This means that a large fraction of the water content will be located within the voids between the polyelectrolyte chains. Recent experiments have also been used to estimate the water content of polyelectrolyte–surfactant complexes,^29,102^ but none of the combinations are similar enough to directly compare to our simulation results.

### Friction of Hair with CGG–SDS Complexes

The final systems from the sequential adsorption simulations are replicated and flipped, and a water/SDS bath is added in the *x*-direction for the squeeze-out simulations. For the squeeze-out simulations at 10 MPa, the final contact thickness is approximately 6 nm for the CGG–SDS complex between both virgin and bleached hair surfaces, as shown in the Supporting Information (Figure S5). The effect of CGG–SDS complexes on friction is investigated with NEMD simulations using the configurations obtained from the squeeze-out simulations with the water/SDS bath removed and periodic surfaces restored in the *x*-direction.^4^ The water and SDS surface coverages used in the NEMD simulations are summarized in Table 1. On virgin hair at σ = 10 MPa, the surface coverages inside the contact equate to ρ_w_ = 16.9 nm^–2^ for water and ρ_SDS_ = 1.11 nm^–2^ for SDS. The SDS coverage is somewhat higher than that adsorbed on virgin hair (1.0 nm^–2^) at the same concentration (*c*_SDS_ = 664 mM) prior to squeeze out.^11^ This is due to the electrostatic attraction between the anionic SDS headgroup beads and nonsurface-bound CGG cationic beads, which are stronger than the hydrophobic interactions between the SDS tailgroup beads and the surface-grafted 18-MEA tailgroup beads. The presence of the anionic surfactant is responsible for an increase in water content compared with both untreated (ρ_w_ = 6.5 nm^–2^) and CGG-treated virgin hair (ρ_w_ = 10.4 nm^–2^). The ratio between anionic sites from SDS and cationic sites on CGG is approximately 1.37 on virgin hair at σ = 10 MPa, which is comparable to experiments.^50^ The excess of SDS molecules results from the adsorption of intact micelles docking during the adsorption stage, and this is retained during squeeze-out at σ = 10 MPa. On medium bleached hair surfaces, the adsorption densities of CGG are the same as for virgin hair. Squeeze-out simulations at σ = 10 MPa on medium bleached hair lead to a surface coverage of ρ_w_ = 20.9 nm^–2^ for water, which moderately increased compared to bleached hair without CGG treatment (ρ_w_ = 16.3 nm^–2^).^4^ For SDS, a surface coverage of ρ_SDS_ = 0.87 nm^–2^ is observed, which corresponds to a moderate excess (ratio of 1.07) of anionic charges compared to cationic sites from CGG. The SDS coverage is higher than that adsorbed on medium bleached hair (0.7 nm^–2^) at the same concentration (*c*_SDS_ = 664 mM) prior to squeeze out.^11^ Thus, CGG locks SDS molecules inside the contact at high pressures through electrostatic interactions.

**Figure 4:**
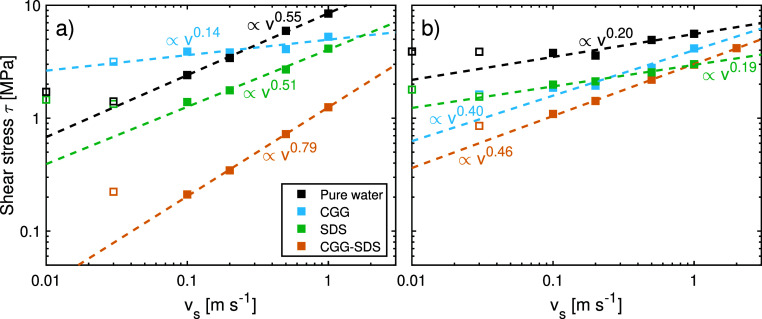
Shear stress as a function of the sliding velocity *v*_s_ at σ = 10 MPa for (a) virgin and (b) medium bleached hair surfaces with CGG and SDS. The shear stress profiles for CGG-only, SDS-only^11^ and pure water^4^ contacts are shown for comparison. Dashed lines are power-law fits to the data.^103^

First, NEMD simulations of the compressed contact at a normal load of σ = 10 MPa were conducted at a range of sliding velocities *v*_s_. Figure 4 shows the shear stress as a function of the sliding velocity in a double-logarithmic representation. As is commonly noted for boundary-lubricated systems,^104^ the shear stress increases with sliding velocity for all of the systems. At sufficiently high sliding velocities, the logarithm of the shear stress obeys power-law scaling. This behavior has previously been observed for self-mated polymer and hydrogel friction in both experiments and simulations.^103^ For CGG–SDS adsorbed on virgin hair, we find a dependency of ∝*v*_s_^0.79^, and ∝*v*_s_^0.46^ for medium bleached hair. These values for polyelectrolyte–surfactant complexes are higher than those for contacts with pure water, which show a weaker velocity dependence. For the pure water systems, we previously attributed this power-law scaling to a shear-augmented thermal activation behavior in the contacts for virgin and bleached hair.^4^ The CGG–SDS complex shear stress is also lower than that observed for pure SDS^11^ and pure CGG (Figure S6). Thus, there is synergism between the CGG and SDS in terms of their lubrication performance.^105^

On virgin hair, the CGG systems increase the shear stress at low sliding velocity compared to pure water but decrease it at high sliding velocity. On bleached hair, CGG reduces the shear stress at all studied sliding velocities. A full analysis of the friction of the CGG only systems is given in the Supporting Information (Section 4). Overall, CGG–SDS systems render a lower shear stress than systems with the respective single component, CGG or SDS, which suggests that synergistic effects are relevant.^105^

The CGG–SDS complexes deposited on the surfaces provide low shear stress on both virgin (τ = 0.2–1.2 MPa) and medium bleached hair surfaces (τ = 1.1–2.9 MPa) at all considered sliding velocities. The shear stress is consistently lower than in the pure water contacts^4^ as well as values reported for cationic surfactants on virgin (τ = 0.4–8.0 MPa) and medium bleached (τ = 1.6–7.8 MPa) hair surfaces under identical conditions, *v*_s_ = 0.03–1 m s^–1^ and σ = 10 MPa.^11^ Thus, friction is lower than both the pure CGG and pure SDS cases, suggesting that the combination of cationic polymers and anionic surfactants provides synergistic friction behavior.^105^ The presence of surfactants is crucial to obtaining low friction, which is consistent with previous experiments of other polyelectrolyte-SDS complexes.^106^

**Figure 5:**
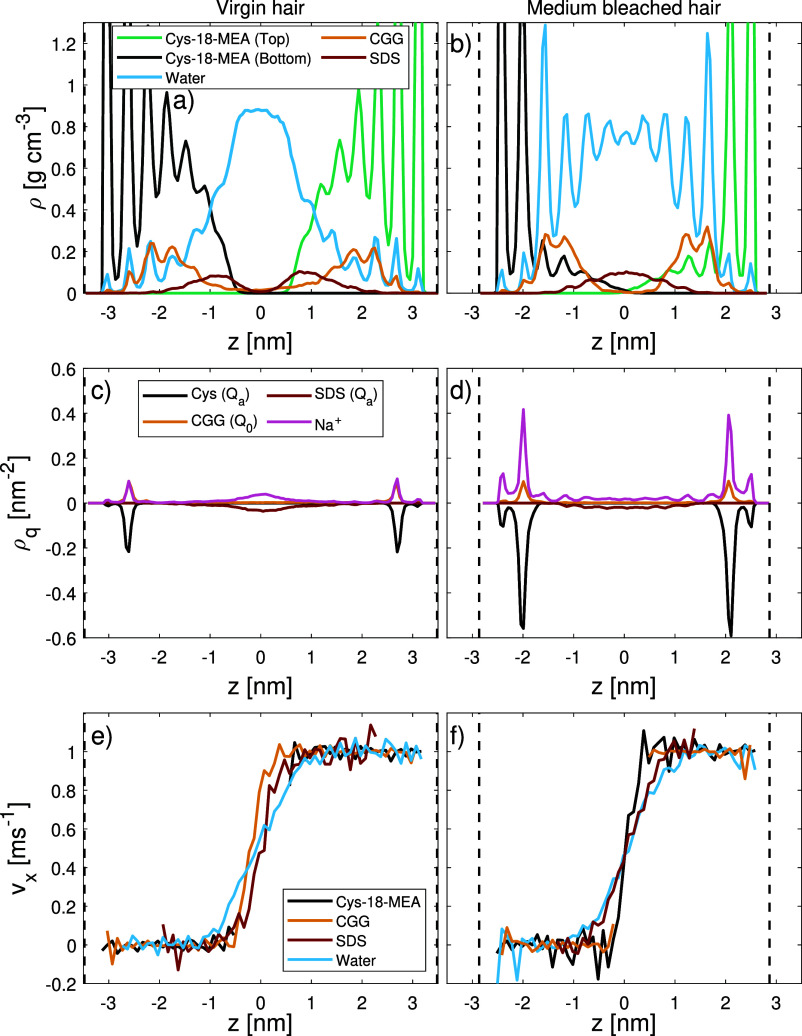
Through-film mass density, ρ (a,b), charge density, ρ_q_ (c,d), and velocity, *v*_*x*_ (e,f) profiles during sliding at σ = 10 MPa and *v*_s_ = 1 m s^–1^ for virgin (a,c,e) and medium bleached (b,d,f) hair.

Figure 5 shows the mass density, charge density, and velocity profiles for the various components at the contact, including the covalently bound 18-MEA layers at *v*_s_ = 1 m s^–1^. As reported in our previous studies,^4,11^ we find the mass and charge density profiles are insensitive to the sliding speed. For virgin hair, a clear separation between the 18-MEA monolayers on the opposing surfaces is apparent at σ = 10 MPa and there is also a gap between the adsorbed CGG layers on each surface. The adsorbed SDS micelles break up during the squeeze-out stage and reorient to form a reverse-bilayer structure,^107^ which is evident from the distribution of the polar headgroup in the charge density profile in Figure 5c. A transition from micelles to partially flipped bilayers under high loads has also been observed in previous experiments of cationic surfactants between mica surfaces.^108^ The charge density profile for virgin hair reveals that the anionic groups on SDS are predominantly oriented toward the center of the bilayer. Such a configuration is only stable due to charge neutralization by the sodium counterions, which are localized at the middle of the contact. The formation of the reverse-bilayer structure can be explained by hydrophobic adsorption of the SDS tailgroups onto the 18-MEA monolayer. The water beads are concentrated mostly in the center of the contact and retain a disordered structure. The increased water uptake in CGG–SDS treated systems compared to bare virgin hair is expected to be closely coupled to the reverse-bilayer of SDS that renders the surfaces more hydrophilic. The velocity profiles for virgin hair suggest that only the central ∼1 nm of the film is sheared, which contains almost exclusively water and SDS. The reduction in interdigitation due to increased levels of retained water and SDS compared to untreated hair surfaces is expected to contribute to the lower friction.

On medium bleached hair at σ = 10 MPa (Figure 5b), the 18-MEA monolayers on the two opposing surfaces are slightly interdigitated. There is also some overlap of the strongly adsorbed CGG layers on the opposing surfaces. SDS is concentrated at the contact center in a single layer, and the charge density profile (Figure 5d) reveals a more disordered structure of SDS compared to the virgin hair case. A strongly layered water film (*d* ≈ 4 nm) provides further surface separation. We previously observed water layering in NEMD simulations of bare damaged hair surface mimics which was attributed to the anionic surface charges.^4^ Similar ordering has been observed in previous experiments of water under nanoscale confinement between cationic surfactant monolayers.^109^ The relatively low degree of CGG adsorption in this work does not completely suppress this layering despite introducing additional disorder to the system. The presence of somewhat less ordered water at the contact center is expected to be beneficial for lubrication rather than increasing friction, as observed in pure water contacts on bleached hair.^4^ We expect that the presence of SDS at the bleached hair interface also leads to further friction reductions due to its repulsive effect and increase in physical separation, which seems to outweigh any dissipative contributions under the present conditions. Friction is further reduced by 18% at *v*_s_ = 0.1 m s^–1^ compared to the previous pure CGG simulations. Wu et al.^23^ observed similar experimental friction reductions of CGG mixed with SDS compared to the pure CGG case. Hössel et al.^84^ also reported wet combing work reductions between 10 and 50% for SLES shampoo formulations containing low CGG concentrations.

Overall, our results suggest that CGG promotes increased electrostatic SDS adsorption on the surfaces. This leads to increased surface separation at a given pressure. Therefore, friction observed in the CGG–SDS systems primarily arises from viscous dissipation in the fluid SDS/water layer at the sheared interface rather than interdigitation between the rigid adsorbed CGG or grafted 18-MEA layers.

#### Effect of Varying Pressure

**Figure 6:**
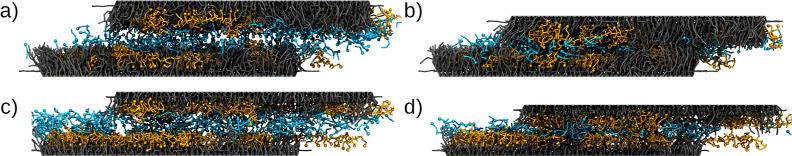
Snapshots of hair contacts with CGG (orange) and SDS (cyan) for virgin (a,b) and medium bleached hair (c,d) at normal stresses of σ = 10 MPa (left) and σ = 50 MPa (right) at *v*_s_ = 0.1 m s^–1^. Water and counterions are not shown for clarity.

**Figure 7:**
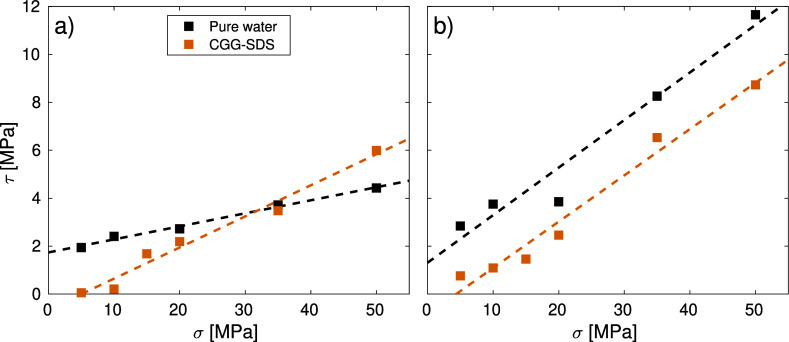
Shear stress as a function of the normal load at constant sliding velocity of *v*_s_ = 0.1 m s^–1^ for (a) virgin and (b) medium bleached hair surfaces with CGG (DS = 100%) and SDS at variable composition and for pure water cases. For comparison, the shear stress for pure water contacts^4^ is shown for comparison. Dashed lines are linear fits to the extended Amontons–Coulomb equation.

The change in the shear stress with normal stress is shown in Figure 7 for a constant sliding velocity of *v*_s_ = 0.1 m s^–1^. Snapshots of virgin and bleached hair contacts at a baseline normal stress of 10 MPa and a maximum normal stress of 50 MPa are shown in Figure 6. On both virgin and bleached hair, the shear stress for the CGG–SDS lubricated systems is very low (τ < 1 MPa) at low normal stress (σ ≤ 10 MPa), before increasing linearly with normal stress. This stepwise increase in shear stress with normal stress is consistent with previous experiments using a different polyelectrolyte-SDS complex to lubricate mica surfaces.^106^ This means that the highest lubrication performance of CGG–SDS is at low normal stresses (≤10 MPa). This regime is probably the most relevant to hair manipulation during touching, brushing, and combing.^4^

**Figure 8:**
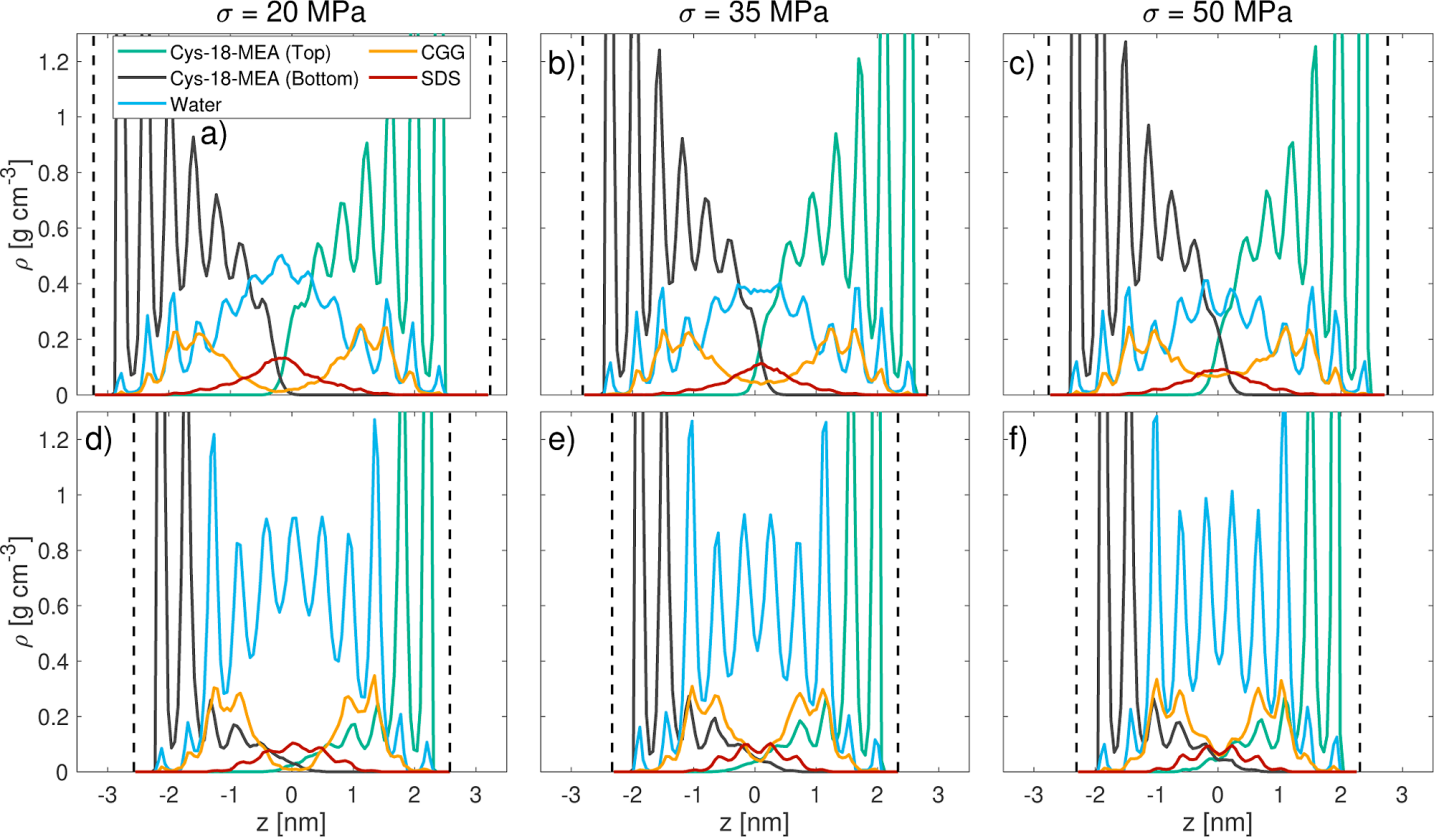
Through-film mass density profiles from NEMD of different normal stresses σ = 20–50 MPa on virgin (a–c) and medium bleached hair (d–f) at *v*_s_ = 0.1 m s^–1^.

The extended Amontons friction law τ = μ·σ + τ_0_ was applied to the data, where μ is the friction coefficient and τ_0_ is the Derjaguin offset.^4^ The mass density profiles of the contact are shown in Figure 8 for σ = 20–50 MPa (σ = 15 MPa omitted due to a lack of space) and in Figure 5a,b for the baseline case at σ = 10 MPa. The corresponding charge density profiles are shown in the Supporting Information (Figure S9).

For all systems and conditions shown in Figure 5, the shear stress is higher on medium bleached hair than on virgin hair. However, the shear stress is more similar for the bleached and virgin hair when they are lubricated by CGG–SDS complex rather than water. On virgin hair, the shear stress, τ, of the CGG–SDS complex strongly increases with the normal stress, σ. At low normal stress (σ < 35 MPa), the CGG–SDS-lubricated contact shows lower shear stress than pure water; however, the trend reverses at higher normal stress. Thus, the CGG–SDS complex reduces the Derjaguin offset, but increases the friction coefficient compared to pure water. On bleached hair, the CGG–SDS complex reduces the shear stress by a similar amount at all normal stress values simulated. This means that CGG–SDS decreases the Derjaguin offset and maintains a similar friction coefficient to when the contact is lubricated by pure water. Thus, the main benefit of the CGG–SDS complex is a reduction in the Derjaguin offset, i.e., the shear stress at low normal stress, which is mainly due to adhesion.^4^ Indeed, previous experimental studies have shown that complexes formed from cationic polyelectrolytes and anionic surfactants can significantly reduce adhesion between negatively charged silica surfaces.^99^

The mass density profiles in Figure 8 suggest that the 18-MEA of the opposite hair surfaces becomes more interdigitated at higher normal loads. This is particularly evident on virgin hair model surfaces. This mechanical effect due to stronger compression is accompanied by a decrease of SDS and water at the interface from squeeze-out. On virgin hair, the SDS bilayer structure observed at σ = 10 MPa also vanishes at higher loads, leading to a more disordered distribution of individual SDS molecules at the interface. The removal of the fluid SDS and water layers and increase in CGG and 18-MEA interdigitation at higher normal stress lead to strongly increasing shear stress and thus relatively high friction coefficients. We expect that higher CGG surface coverages will lead to increased SDS trapping and thus lower friction. Moreover, higher CGG surface coverages and lower CGG charge densities may enable more tails and loops to form, which could be important to lubrication.^84^ Finally, overcompensation of the surface charge may enable increased swelling when SDS micelles are added, leading to a further increase in the number of tails and loops. These factors will be investigated in a future study.

Future extensions of this work could also investigate the adsorption, squeeze-out and friction of phase-separated polyelectrolyte–surfactant networks, or coacervates,^53,54^ to hair surfaces, as has been studied experimentally.^23,26^ However, additional method development is required to consider microscale aggregate sizes since the current squeeze-out setup is limited to systems in which all polyelectrolytes strongly adsorb to the surfaces, so they remain within the contact. The framework presented in this study could be applied to virtually screen the lubrication performance of a wide range of hair care formulations on biomimetic surfaces. By changing the surface model, the methodology can be readily extended to investigate other formulated products that contain polymer–surfactant complexes, such as in fabric softeners^46^ or for drug delivery.^47^

## Conclusions

We investigated the lubrication performance of adsorbed complexes formed from cationic polyelectrolytes and anionic surfactants between two hair surfaces using coarse-grained NEMD simulations. We have developed new MARTINI 2 parameters of guar hydroxypropyltrimonium chloride by employing existing mappings and bead types for saccharides and fine-tuning the bond and angle interactions using Boltzmann inversion from atomistic simulations with the OPLS force field. The new MARTINI 2 parameters are able to reproduce the probability distribution functions for the relevant bonds and angles from the atomistic simulations. The power-law scaling of the guar radius of gyration with molecular weight seen experimentally is also reproduced with the new MARTINI 2 parameters.

In the MD simulations, CGG oligomers were first deposited onto biomimetic surfaces representing virgin and medium bleached hair at an adsorption density similar to that measured experimentally for another cationic polysaccharide. Due to computational constraints, we limit ourselves to the study of relatively short CGG polymers with high charge density. The cationic groups on CGG strongly adsorb to the anionic groups on the hair surfaces, leading to mostly flat train conformations. This observation agrees with experiments for high charge density cationic polyelectrolytes on anionic-functionalized surfaces. Next, we introduced anionic surfactant SDS to the hair surfaces containing adsorbed CGG. SDS predominantly adsorbed as intact cylindrical micelles, binding to the cationic sites on CGG that were not attached to the anionic groups on the hair surfaces. We observe some swelling of the CGG layers upon addition of SDS, but far less than that seen experimentally. This discrepancy might be due to the relatively high charge density and small degree of polymerization of the polyelectrolytes used in our simulations. Another factor is the relatively low polyelectrolyte surface coverage studied, which means that the negative surface charge is not overcompensated by the CGG.

We then performed squeeze-out and NEMD simulations between two hair surfaces containing adsorbed CGG and SDS. Pure CGG was found to reduce shear stress at high sliding velocity compared to pure water but increased shear stress and stick–slip amplitudes at low sliding velocity. This was likely caused by the heterogeneous distribution of strongly adsorbed CGG and the resulting oscillatory interactions of hydrophilic CGG sites sliding relative to each other across the otherwise hydrophobic 18-MEA monolayers. On bleached hair, pure CGG reduced friction considerably compared to untreated contacts at all sliding velocities.

The CGG–SDS complexes significantly reduced the shear stress on both virgin and medium bleached hair compared to water, pure CGG, and pure SDS across all the sliding velocities considered. Thus, there is a synergistic lubrication performance for the CGG–SDS complex compared to CGG and SDS. CGG strongly adsorbs to the hair surface, neutralizing the surface anionic groups and providing cationic sites for the adsorption of SDS micelles. The adsorbed SDS micelles provide a direct lubrication effect, while also leading to a hydrophilic-induced increase of water trapped within the contact. At low normal stress, friction is dominated by viscous dissipation in the fluid SDS and water interfacial layer. With increasing normal stress during squeeze-out, a larger fraction of SDS and water are depleted from the contact. This means that the highest lubrication performance of the CGG–SDS is at low normal stresses (≤10 MPa). This regime is probably the most relevant to hair manipulation during touching, brushing, and combing. On virgin hair, the shear stress of the CGG–SDS complex eventually exceeds that of pure water at high (>30 MPa) normal stress. Thus, CGG–SDS leads to a decrease in Derjaguin offset but an increase in friction coefficient on virgin hair. On bleached hair, the shear stress with CGG–SDS remains lower than for water under all studied normal stresses. In this case, CGG–SDS leads to a reduction in Derjaguin offset and a similar friction coefficient as for water. The friction coefficient becomes insensitive to hair damage for CGG–SDS lubricated contacts.

The computational framework presented in this work is expected to be useful for benchmarking a wide range of polymers and surfactants for their relative lubrication performance on hair surfaces. It can also be readily extended to screen the performance of other formulations where polyelectrolyte–surfactant complexes play an important role.

## Supporting Information Available

Details of CGG MARTINI force field parameters, additional CGG parameter validation and additional figures on the analysis of NEMD simulations of CGG and CGG–SDS contacts. (PDF)
Exemplary LAMMPS data-files and input scripts for sequential adsorption, squeeze-out, and NEMD simulations and CGG MARTINI parameters are available in a dedicated GitHub repository which can be found on Zenodo (DOI:10.5281/zenodo.10610684)
