# Supplementary material for: Boundary Lubrication Performance of Polyelectrolyte–Surfactant Complexes on Biomimetic Surfaces
Source: Langmuir. 2024 Apr 4;40(15):7933–46. doi: 10.1021/acs.langmuir.3c03737 (PMC11025133; doi:10.1021/acs.langmuir.3c03737)
Supplement: Supplementary file 1 — la3c03737_si_001.pdf [file la3c03737_si_001.pdf]

Supporting Information for: Boundary lubrication performance of polyelectrolyte-surfactant complexes on biomimetic surfaces

Erik Weiand,[*,†,‡,¶] Peter H. Koenig,[§] Francisco Rodriguez-Ropero,[§] Yuri Roiter,[§] Stefano Angioletti-Uberti,[‖,‡,¶] Daniele Dini,[†,‡,¶] and James P. Ewen[*,†,‡,¶]

†*Department of Mechanical Engineering, Imperial College London, South Kensington Campus, SW7 2AZ London, U.K.*

‡*Institute of Molecular Science and Engineering, Imperial College London, South Kensington Campus, SW7 2AZ London, U.K.*

¶*Thomas Young Centre for the Theory and Simulation of Materials, Imperial College London, South Kensington Campus, SW7 2AZ London, U.K.*

§*Corporate Functions Analytical and Data & Modeling Sciences, Mason Business Center, The Procter and Gamble Company, Mason, 45040 Ohio, U.S.A.*

‖*Department of Materials, Imperial College London, South Kensington Campus, SW7 2AZ London, U.K.*

E-mail: erik.weiand19@imperial.ac.uk; j.ewen@imperial.ac.uk

MARTINI force field parameters

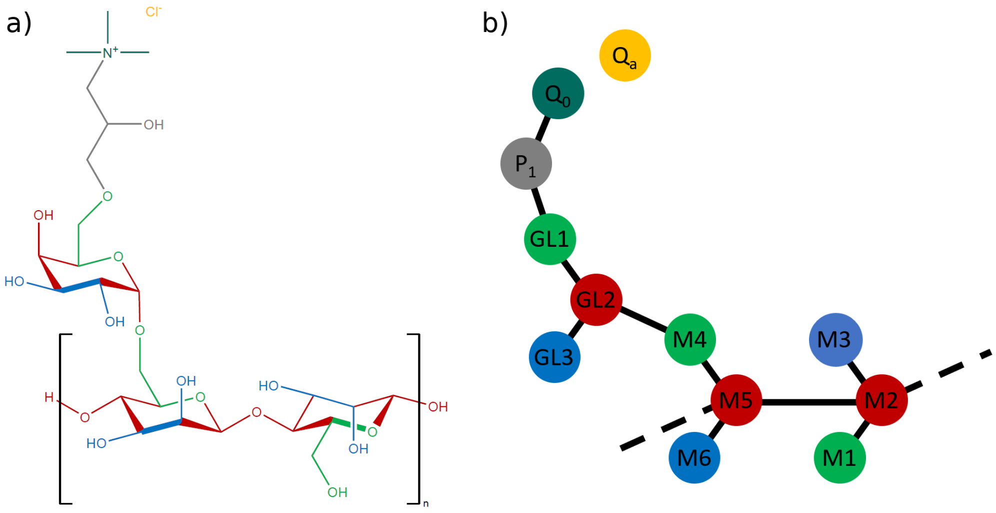

Figure S1: a) chemical structure of cationic guar gum (CGG) or guar hydroxypropyltrimonium chloride and b) the corresponding coarse-grained architecture.

Table S1: MARTINI 2 bead types definitions.

**Bead Types**				
Building block	Bead name	MARTINI type	Charge, q [e]	Molar mass, M [kg/kmol]
Chloride counterion	Q_a	Q_a	-1.0	72.0
Quaternary amine	Q_0	Q_0	$+1.0$	72.0
Hydroxypropyl	P_1	P_1	-	72.0
Galactose-I	GL_1	P_1	-	72.0
Galactose-II	GL_2	SP_2	-	54.0
Galactose-III	GL_3	P_4	-	72.0
Mannose-I	M_1, M_4	P_1	-	72.0
Mannose-II	M_2, M_5	SP_2	-	54.0
Mannose-III	M_3, M_6	P_4	-	72.0

There are four main types of interaction sites in MARTINI 2, which are differentiated: polar (P), nonpolar (N), apolar (C), and charged (Q).[1] The special class of ring-type particles (S) have a reduced interaction strengths. Each particle type has a number of sub-types, which allows for a more accurate representation of the chemical nature of the underlying atomic structure.[1]

Within a main type, subtypes are either distinguished by a letter denoting the hydrogen-bonding capabilities, (d) donor, (a) acceptor, (da) both, or (0) none, or by a number indicating the degree of polarity (from 1, low polarity, to 5, high polarity).[1] The MARTINI 2 mapping we used for CGG was adapted from that proposed by Shivgan et al.,[2] which was modified from the original mapping due to López et al.[3]

Bond and angle interaction parameters for CGG are derived from iterative Boltzmann inversionn (IBI) from atomistic simulations using PyCGTOOL,[4] as described in the main text.

Table S2: Pol-MARTINI 2 non-bonded Lennard-Jones parameters for CGG.

**LJ interactions**				
Bead A	Bead B	Interaction strength	ε [kcal/mol]	σ [Å]
Q_0	Q_0	IV	0.8361	4.7
	P_1	II	1.0750	4.7
	GL_1	II	1.0750	4.7
	GL_2	I	1.1945	4.7
	GL_3	O	1.3378	4.7
	M_1 / M_4	II	1.0750	4.7
	M_2 / M_5	I	1.1945	4.7
	M_3 / M_6	O	1.3378	4.7
P_1	P_1	II	1.0750	4.7
	GL_1	II	1.0750	4.7
	GL_2	II	1.0750	4.7
	GL_3	II	1.0750	4.7
	M_1 / M_4	II	1.0750	4.7
	M_2 / M_5	II	1.0750	4.7
	M_3 / M_6	II	1.0750	4.7
GL_1	GL_1	II	1.0750	4.7
	GL_2	II	1.0750	4.7
	GL_3	II	1.0750	4.7
	M_1 / M_4	II	1.0750	4.7
	M_2 / M_5	II	1.0750	4.7
	M_3 / M_6	II	1.0750	4.7
GL_2	GL_2	S-II	0.8063	4.3
	GL_3	II	1.0750	4.7
	M_1 / M_4	II	1.0750	4.7
	M_2 / M_5	S-II	0.8063	4.3
	M_3 / M_6	II	1.0750	4.7
GL_3	GL_3	I	1.1945	4.7
	M_1 / M_4	II	1.0750	4.7
	M_2 / M_5	II	1.0750	4.7
	M_3 / M_6	I	1.1945	4.7
M_1 / M_4	M_1 / M_4	II	1.0750	4.7
	M_2 / M_5	II	1.0750	4.7
	M_3 / M_6	II	1.0750	4.7
M_2 / M_5	M_2 / M_5	S-II	0.8063	4.3
	M_3 / M_6	II	1.0750	4.7
M_3 / M_6	M_3 / M_6	I	1.1945	4.7

Table S3: MARTINI 2 bond parameters.

**Bonds**		$E_\mathrm{b} = K_\mathrm{b}\,(r - r_0)^2$	
Bead A	Bead B	$\mathrm{K_b}$ [kcal/mol/Å^2]	$\mathrm{r_0}$ [Å]
Q_0	P_1	75	3.42
P_1	GL_1	27	3.90
GL_1	GL_2	12	2.41
GL_2	GL_3	15	2.41
GL_2	M_4	16	5.20
M_4	M_5	88	2.22
M_5	M_6	69	2.09
M_5	M_2	64	5.66
M_1	M_2	79	2.41
M_2	M_3	27	2.30
M_2	M_5'	50	4.54

Table S4: MARTINI 2 angle parameters.

**Angles**			$E_\theta = K_\theta\,[\cos(\theta) - \cos(\theta_0)]^2$	
Bead A	Bead B	Bead C	K_θ [kcal/mol]	θ_0 [°]
Q_0	P_1	GL_1	22	108
P_1	GL_1	GL_2	558	162
GL_1	GL_2	GL_3	63	141
GL_1	GL_2	M_4	46	75
GL_3	GL_2	M_4	79	67
GL_2	M_4	M_5	87	122
M_4	M_5	M_6	832	161
M_4	M_5	M_2	90	99
M_6	M_5	M_2	156	96
M_5	M_2	M_1	243	74
M_5	M_2	M_3	379	58
M_1	M_2	M_3	128	126
M_4	M_5	M_6	832	161
M_1	M_2	M_5'	194	89
M_3	M_2	M_5'	175	123
M_5	M_2	M_5'	15	154
M_2	M_5'	M_2'	30	162

Guar gum parametrization validation

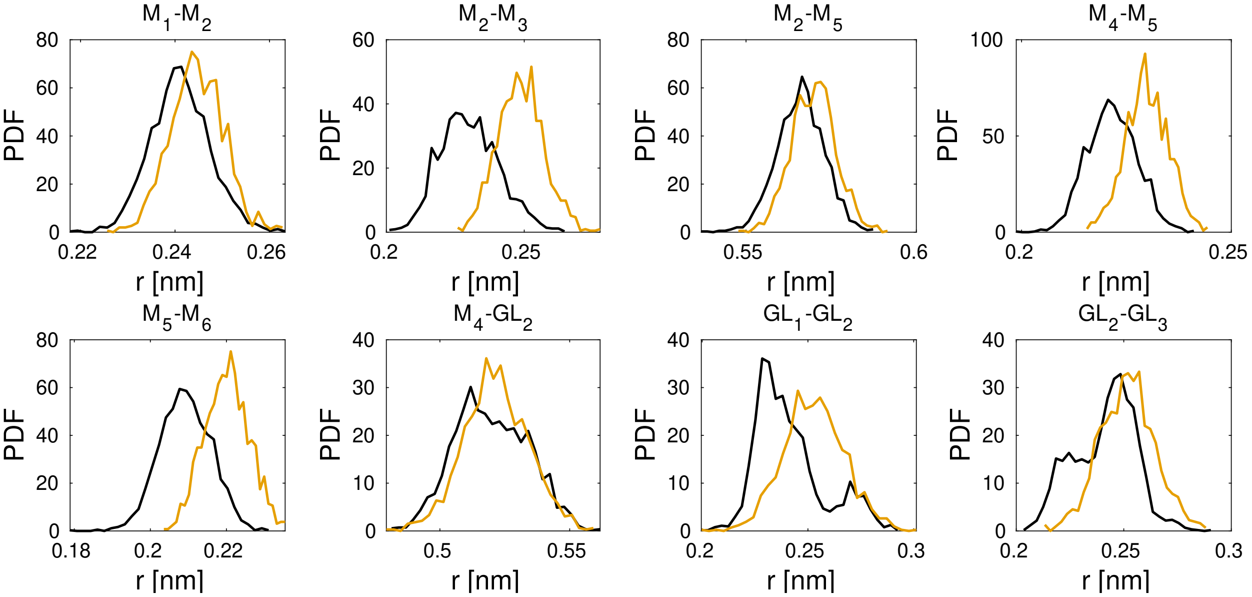

Figure S2: Guar gum bond distributions from atomistic (AA-MD) and coarse-grained simulations.

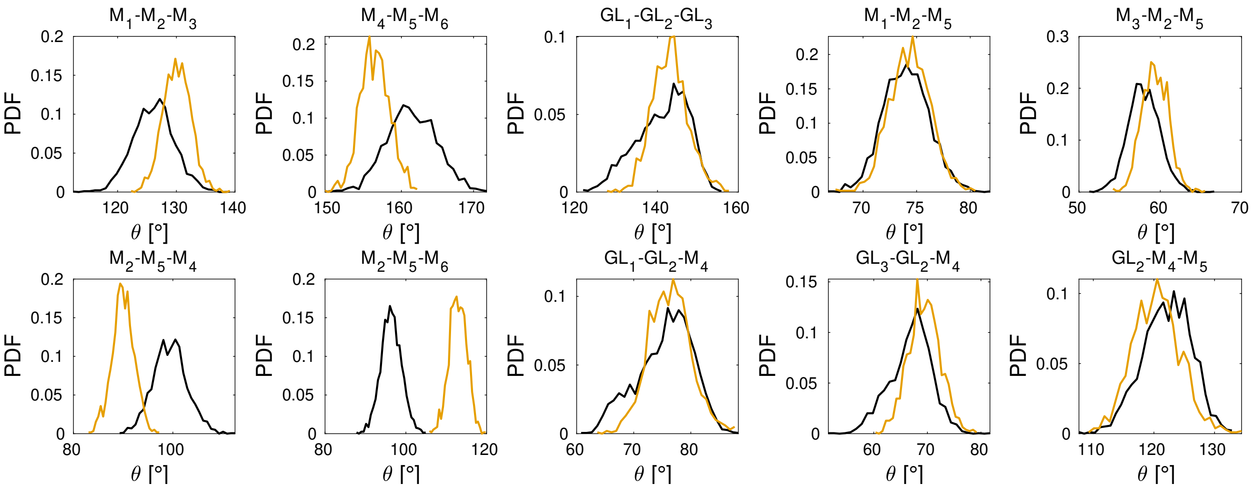

Figure S3: Guar gum angle distributions from atomistic (AA-MD) and coarse-grained simulations.

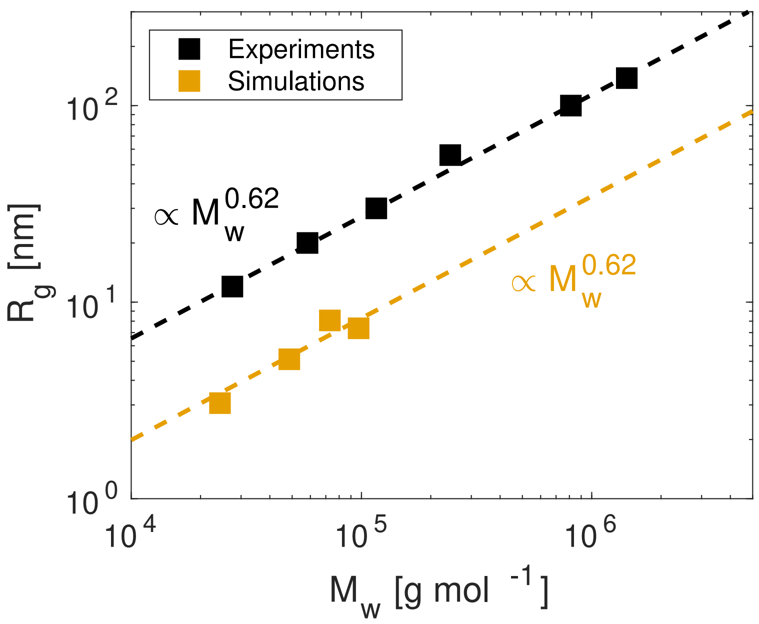

Figure S4: Guar gum radius of gyration as a function of molecular weight from CG-MD simulations and from experiments from Ref.[5]

CGG-SDS squeeze-out

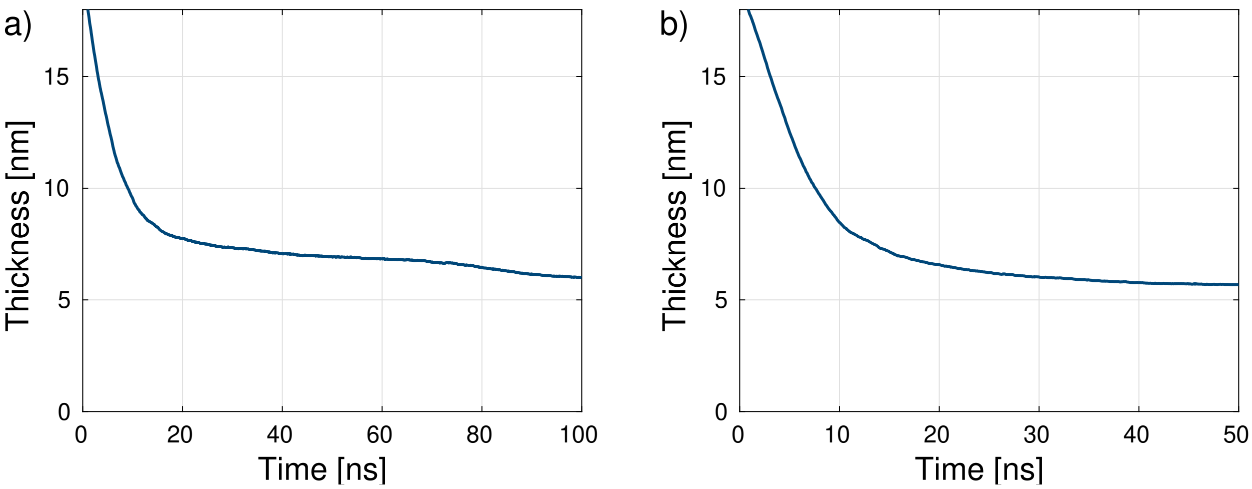

Figure S5: Temporal evolution of the contact thickness during squeeze-out at $\sigma = 10$ MPa with CGG and SDS on a) virgin and b) medium bleached hair model hair surfaces.

Friction with pure CGG

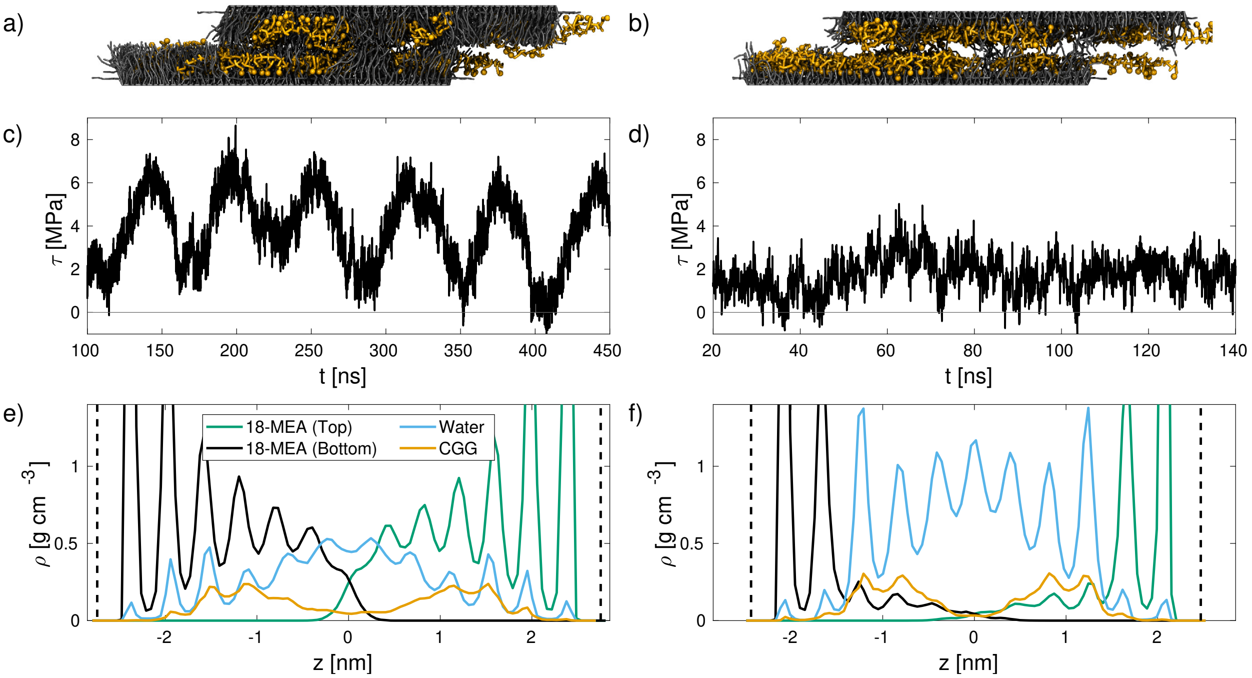

Figure S6: Snapshots of NEMD simulations of a) virgin and b) medium bleached hair surfaces with CGG at $\Gamma = 0.04$ μg m^{-2}, sliding velocity of $v_s = 0.1$ ms^{-1} and a contact pressure of $\sigma = 10$ MPa. Respective shear stress profiles as a function of time are shown in c)-d). Signals are only shown from the point when a dynamic equilibrium has been reached. Time-averaged mass density profiles for e) virgin and f) medium bleached hair are shown for for surface lipids (only up to $\pm$1 nm from the contact center for clarity), guar and water within the contact.

Prior to introducing anionic SDS surfactants into the contact, the conditioning performance of pure CGG with high charge density (DS = 100%) was investigated in NEMD simulations at a normal load of $\sigma = 10$ MPa and sliding velocity of $v_s = 0.1$ ms^{-1}. None of the CGG molecules were removed from the contact during the squeeze-out stage due to the formation of strong ionic bonds with the surfaces. The water trapped within the contact at $\sigma = 10$ MPa increases by around 60% from a surface coverage of $\rho_{\mathrm{w}} = 6.5$ nm^{-2} for un-

treated virgin hair,[6] to $\rho_{\mathrm{w}} = 10.4$ nm^{-2} for virgin hair with adsorbed CGG. On the bleached hair surfaces, the water coverage increases by approximately 10% from $\rho_{\mathrm{w}} = 16.3$ nm^{-2} for untreated hair,[6] to $\rho_{\mathrm{w}} = 18.1$ nm^{-2} with CGG adsorbed. The observed increase in water beads trapped in the contact can be explained by the relatively high charge density of the CGG, which means that not all of the cationic sites bind to the surface, exposing an increased number of hydrophilic sites to the aqueous bulk.

Fig. S6a)-b) shows snapshots of the sliding contact and corresponding shear stress signals for virgin and medium bleached hair in the presence of adsorbed CGG at $v_s = 0.1$ ms^{-1}. The shear stress with CGG on virgin hair, $\tau = 3.9 \pm 1.7$ MPa is significantly increased in comparison to values previously reported on untreated virgin hair surfaces, $\tau = 2.4$ MPa at 10 MPa.[6] For virgin hair, strong periodic oscillations are observed at a characteristic frequency of $f \approx 0.015$ ns^{-1}. Previous investigations of stick-slip of polymers at interfaces revealed a strong correlation of the friction signal amplitude with local displacement of polymers.[7] We checked the root-mean square displacement of the polymers and found no periodic fluctuations of the displacements (Fig. S8), which suggests that polymer dynamics are not directly linked to the oscillations. The time-averaged mass density profiles shown in Fig. S6e)-f) indicate that the interdigitation between opposite surfaces is more pronounced for virgin than for bleached hair. This might explain parts of the higher ob-

served CoF. An increase in stick-slip is however deemed unlikely to be solely caused by interdigitation of the 18-MEA, due to the high coverage of lipids.[8] A fast Fourier transform (FFT) of the topography signal of guar on the respective surfaces revealed a characteristic wavelength of $\kappa = 0.016$ nm^{-1}, which is directly connected to the previously observed stick-slip frequency via the transport velocity $v_s = f/\kappa$. The surface topography distributions and corresponding FFT spectra are shown in Fig. S7 The agreement of the oscillation frequency with the characteristic wavelength suggests that the observed oscillations are promoted by the local heterogeneous distribution of guar in the direction of sliding x on each surface. The high charge density on the studied CGG molecules might further contribute to rigid, heterogeneous distribution of polymers on the respective surface which is resistant to sliding.

Our analysis reveals that the studied levels of CGG adsorption do not contribute to favorable friction reductions on virgin hair at relatively low sliding velocities. Previous NEMD simulations with cationic surfactants suggest that friction forces are significantly reduced ($\mu = 0.14$) compared to both CGG-treated and untreated virgin hair surfaces at the same load and sliding conditions.[9] Miyamoto et al.[10] also reported high stick-slip amplitudes for damaged hair treated with certain shampoo formulations but attributed this to entire layers of adsorbed coacervates being sheared off the surface during sliding. An increase in CGG adsorption density further might be beneficial

for effectively reducing friction.

Interestingly, no significant periodic fluctuations in the friction signal are observed for pure guar on medium bleached hair. On bleached hair, guar provides considerable reductions in friction ($\tau = 1.7 \pm 0.9$ MPa) compared to the bare wet bleached contact ($\tau = 3.8$ MPa). The corresponding mass density profiles reveal little surface interdigitation between the remaining 18-MEA lipids. The considerable friction reductions might be a result of the guar being able to distribute more evenly on the lipid-depleted bleached model surfaces. On bleached surfaces with adsorbed guar, the coarse-grained water film shows moderate ordering in the surface-normal direction. This is consistent with previous NEMD simulations on unconditioned bleached model hair surfaces.[6]

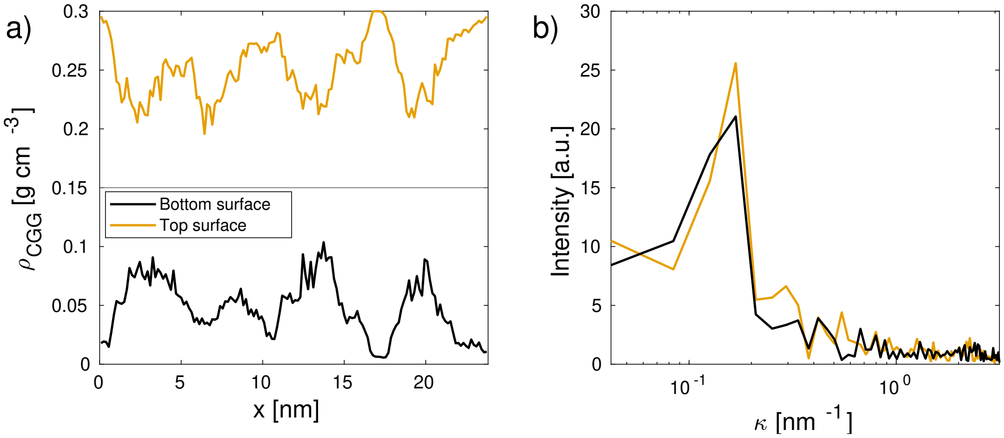

Figure S7: a) Surface topography of pure CGG on virgin hair surfaces in the direction of sliding, x. The averaged mass density signal for the upper surface is flipped and shifted by $\Delta\rho = 0.3$ g cm^{-3} for clarity. The characteristic wavelength of the lateral guar distribution, $f' = 0.016$ nm^{-1} is revealed from b) a fast Fourier transform (FFT) of the topography signals.

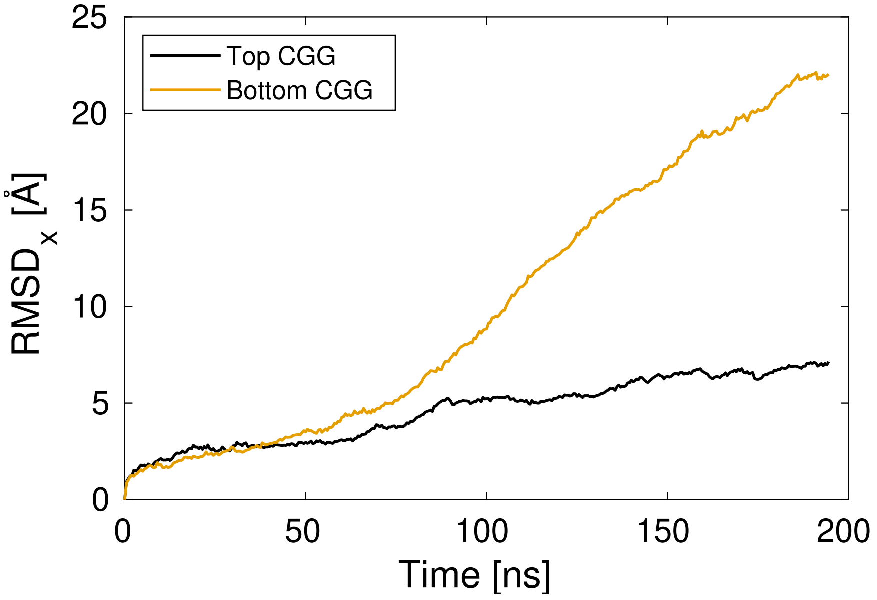

Figure S8: Temporal evolution of the root-mean-square displacement (RMSD) of CGG in virgin hair contacts in the direction of sliding at $\sigma = 10$ MPa and $v_s = 0.1$ ms^{-1}. Different curves show the polymer displacement relative to the respective surface they are initially adsorbed to. Around $t = 50$ ns, a single guar molecule detaches from the bottom hair surface and is dragged in the direction of sliding ($v/v_s \approx 0.5$), which increases the RMSD but does not contribute further to the observed oscillatory behaviour. No periodic fluctuations are evident from the RMSD.

Normal-load variation: charge density profiles

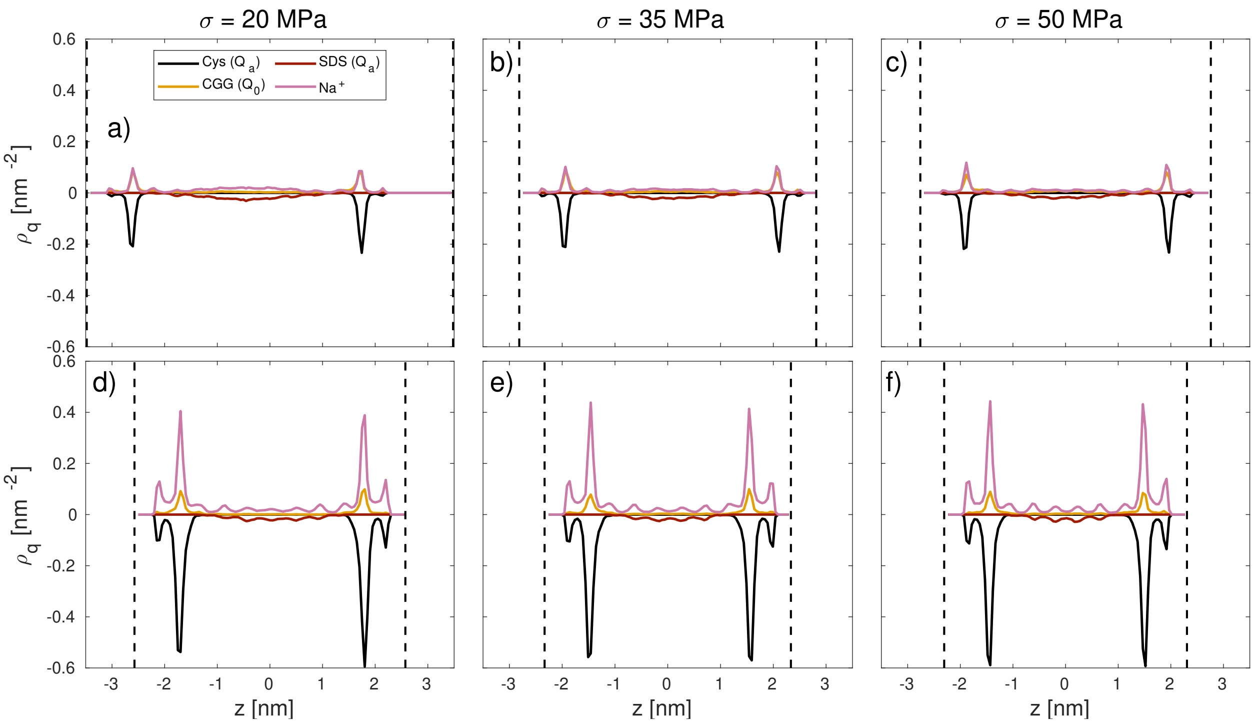

Figure S9: Through-film charge density profiles from NEMD of different normal stresses $\sigma = 20 - 50$ MPa on virgin (a-c) and medium bleached hair (d-f) at $v_s = 0.1$ m s^{-1}.

References

(1) Marrink, S. J.; Risselada, H. J.; Yefimov, S.; Tieleman, D. P.; De Vries, A. H. The MARTINI force field: Coarse grained model for biomolecular simulations. *Journal of Physical Chemistry B* **2007**, *111*, 7812–7824.

(2) Shivgan, A. T.; Marzinek, J. K.; Huber, R. G.; Krah, A.; Henchman, R. H.; Matsudaira, P.; Verma, C. S.; Bond, P. J. Extending the Martini Coarse-Grained Force Field to N-Glycans. *Journal of Chemical Information and Modeling* **2020**, *60*, 3864–3883.

(3) López, C. A.; Rzepiela, A. J.; de Vries, A. H.; Dijkhuizen, L.; Hünenberger, P. H.; Marrink, S. J. Martini coarse-grained force field: Extension to carbohydrates. *Journal of Chemical Theory and Computation* **2009**, *5*, 3195–3210.

(4) Graham, J. A.; Essex, J. W.; Khalid, S. PyCGTOOL: Automated Generation of Coarse-Grained Molecular Dynamics Models from Atomistic Trajectories. *Journal of Chemical Information and Modeling* **2017**, *57*, 650–656.

(5) Liang, H.; Webb, M. A.; Chawathe, M.; Bendejacq, D.; De Pablo, J. J. Understanding the Structure and Rheology of Galactomannan Solutions with Coarse-Grained Modeling. *Macromolecules* **2022**, *56*, 177–187.

(6) Weiand, E.; Ewen, J. P.; Roiter, Y.; Koenig, P. H.; Page, S. H.; Rodriguez-Ropero, F.; Angioletti-Uberti, S.; Dini, D. Nanoscale friction of biomimetic hair surfaces. *Nanoscale* **2023**, *15*, 7086–7104.

(7) Dai, L.; Minn, M.; Satyanarayana, N.; Sinha, S. K.; Tan, V. B. C. Identifying the Mechanisms of Polymer Friction through Molecular Dynamics Simulation. *Langmuir* **2011**, *27*, 14861–14867.

(8) Weiand, E.; Ewen, J. P.; Koenig, P. H.; Roiter, Y.; Page, S. H.; Angioletti-Uberti, S.; Dini, D. Coarse-grained molecular models of the surface of hair. *Soft Matter* **2022**, *18*, 1779–1792.

(9) Weiand, E.; Ewen, J. P.; Roiter, Y.; Koenig, P. H.; Rodriguez-Ropero, F.; Angioletti-Uberti, S.; Dini, D. Effects of Cationic Surfactant Adsorption on the Wettability and Friction of Biomimetic Surfaces. *Physical Chemistry Chemical Physics* **2023**, *25*, 21916–21934.

(10) Miyamoto, T.; Yamazaki, N.; Tomotsuka, A.; Sasahara, H.; Watanabe, S.; Yamada, S. Tribological Properties between Taut Hair Fibers in Wet Conditions: A New Shampoo Formulation for Eliminating Stick-Slip Friction. *Journal of Surfactants and Detergents* **2021**, *24*, 501–510.
